# Lipid Readjustment in *Yarrowia lipolytica* Odd-Chain Fatty Acids Producing Strains

**DOI:** 10.3390/biom12081026

**Published:** 2022-07-25

**Authors:** Sonia Abreu, Young-Kyoung Park, Camilla Pires de Souza, Lea Vidal, Pierre Chaminade, Jean-Marc Nicaud

**Affiliations:** 1 Lipides: Systèmes Analytiques et Biologiques, Université Paris-Saclay, 91400 Orsay, France; sonia.abreu@universite-paris-saclay.fr; 2 Micalis Institute, INRAE, AgroParisTech, Université Paris-Saclay, 78350 Jouy-en-Josas, France; yk16.park@gmail.com (Y.-K.P.); camilla.pires-de-souza@inrae.fr (C.P.d.S.); lea.vidal@inrae.fr (L.V.)

**Keywords:** *Yarrowia lipolytica*, OCFA, lipid profile, lipidome, normal-phase liquid chromatography, metabolic readjustments

## Abstract

*Yarrowia lipolytica* is a promising oleaginous yeast for producing unusual lipids, such as odd-chain fatty acids (OCFA). Their diverse applications and low natural production make OCFA particularly interesting. In recent studies, inhibiting the catabolic pathway of precursor, boosting precursor pools, and optimizing substrate combination greatly improved the production of OCFA in *Y. lipolytica*. We explored the lipid readjustment of OCFA in engineered *Y. lipolytica* strains. NPLC-Corona-CAD^®^ evidenced a time-dependent overproduction of free fatty acids, diglycerides, and phosphatidylcholine (PC) in obese LP compared to obese L. Phosphatidylethanolamine (PE) and phosphatidylinositol, largely overproduced in obese LP at 72 h compared to obese L, vanished at 216 h. The fatty acyls (FAs) composition of glycero- and glycerophospholipids was determined by NPLC-APPI^+^-HRMS from in-source generated monoacylglycerol-like fragment ions. C18:1 and C17:1 were predominant acylglycerols in obese L and obese LP, respectively. Phosphatidic acid, PE, and PC exhibited similar FAs composition but differed in their molecular species distributions. Cardiolipin (CL) is known to contain mostly C18:2 FAs corresponding to the composition in obese L, 50% of C18:2, and 35% of C18:1. In obese LP, both FAs dropped to drop to 20%, and C17:1 were predominant, reaching 55%. We hypothesize that CL-modified composition in obese LPs may alter mitochondrial function and limit lipid production.

## 1. Introduction

In recent years, *Yarrowia lipolytica* has emerged as a favorable chassis for the production of unusual lipids [1,2]. Its oleaginous phenotype, the comprehensive studies on its lipid metabolism, and the advancement of synthetic biology tools provided a huge opportunity to improve the unusual lipid production at high titer, rate, and yield in *Y. lipolytica* [3,4,5,6]. Unusual lipids have received increasing attention as target products of metabolic engineering due to their wide range of applications in industry. Odd chain fatty acids (OCFAs), a type of unusual lipids, have shown various applications in the food, medical, and chemical industries. OCFA can be used as a biomarker of obesity, dietary fiber intake, and the risk of coronary heart disease [7,8,9,10]. *cis*-9-heptadecenoic acid (C17:1) has anti-inflammatory effects and can help treat psoriasis, allergies, and autoimmune diseases [11]. As well as, *cis*-9-heptadecenoic acid is known to have antagonistic activity against powdery mildew (a plant disease) [12]. OCFA and its derivatives have high value as precursors for flavor and fragrance, hydraulic fluids, plasticizers, coatings, and others [12,13,14,15].

Despite their broad applications, the natural production of OCFA is very limited [6,16]. In order to improve the production of OCFA, metabolic engineering and optimization of production conditions were shown in several studies [17]. In recent studies, the production of OCFA in *Y. lipolytica* was greatly improved by inhibiting the catabolic pathway of precursor, boosting precursor pools, and optimizing substrate combination [1,5]. From the engineered strain, 1.87 g/L of OCFA, which was 62% of total lipids, was produced. The composition of fatty acids was also significantly changed, resulting from this genetic engineering. Indeed, in the engineered strain, the ratio of oleic acid (C18:1, main FA produced by *Y. lipolytica*) was only about 15% of total lipids, while more than 45% was heptadecenoic acid (C17:1) [1].

In most studies, the production of lipid is determined by GC-FID analysis after the transesterification of fatty acyl chains [18]. This method is straightforward and robust to quantify the fatty acids and assess the chain length and the degree of saturation. However, this method is not capable of assessing the lipidome, i.e., understanding the distribution of lipids in different classes and the flexibility of the lipidome according to environmental stress or genetic modification. It is known that the lipidome (membrane and metabolic energy storage) is flexible and can be readjusted [19,20]. Therefore, exploring the dynamic modification of lipidome is very important to understand the mechanism of stress response and adaptation of microorganisms. Hein and Hayen investigated the adaptive changes of glycerophospholipids (GP) under various environmental conditions with five phylogenetically different yeasts, including *Y. lipolytica* [21]. It was observed that the GP profiles were significantly different, showing characteristic genetic traits and phylogenetic relationships reflected in the GP profile of the organism. More recently, the lipidome adjustment in *Y. lipolytica* upon different cultivation conditions was explored [22]. Remarkable changes in the composition and the degree of saturation of fatty acids were observed due to the increase in the temperature. Modification of fatty acid composition of some membrane phospholipids was shown at changing pH and temperature. The study reflects the flexibility of lipid composition of *Y. lipolytica* in adaptation to environmental conditions. Apart from the environmental change, the readjustment of lipidome depending on genetic engineering is not explored in *Y. lipolytica* so far.

The recent development of mass spectrometry has paved the way for a more comprehensive and quantitative assessment of lipidome [23]. Through LC-MS and LC with Corona-CAD^®^ detection, notable variations of lipid class composition were observed in modified *Streptomyces* bacteria strains [24,25,26]. In another study, we showed that the FAs distribution of TAGs and PLs could be assessed from the relative intensities of ions [MG+H-H_2_O]^+^ produced in-source using APCI^+^ (or APPI^+^) ionization. This approach allows access to the FAs distribution by lipid class without pretreatment of the lipid extracts, which greatly simplifies the analyses. LC-MS coupling (MS^2^ and MS^3^) was also used for lipid class and molecular species identification [27,28].

In this study, we aimed to explore the lipid readjustment of OCFA in engineered *Y. lipolytica* strains employing the LC-MS method recently developed for lipidomic analysis. First, we assessed the lipid readjustment by comparing chromatographic lipid class analysis profiles obtained by LC with Corona-CAD^®^ detection. Second, we evaluated the lipid rearrangement by comparing the FAs distribution of lipid classes as measured by LC-MS according to the up-to-date methodology.

## 2. Materials and Methods

### 2.1. Strains and Media Composition

*Y. lipolytica* strains, JMY7775 (obese L), and JMY7780 (obese LP) previously constructed in Park et al. [1] were used in this study. Rich medium (YPD) for strain activation and pre-culture was prepared with 1% (*w*/*v*) yeast extract, 2% (*w*/*v*) peptone, and 2% (*w*/*v*) glucose. For lipid accumulation, minimal media (YNBD2P0.5A1) was prepared with 0.17% (*w*/*v*) yeast nitrogen base without amino acids and ammonium sulfate (YNBww), 0.15% (*w*/*v*) NH_4_Cl, 50 mM KH_2_PO_4_–Na_2_HPO_4_ (pH 6.8), 2% (*w*/*v*) glucose, 0.5% (*w*/*v*) propionate and 1% (*w*/*v*) acetate.

### 2.2. Culture Conditions

OCFA biosynthesis experiments were carried out in minimal media under nitrogen-limited conditions, and the cultures were prepared as follows: an initial pre-culture was established by inoculating in 10 mL of YPD medium and grown overnight at 28 °C and 180 rpm. The cells were washed with sterile distilled water and used inoculating to 50 mL of minimal medium in 250 mL Erlenmeyer flasks with initial OD 0.1. The cultures were then incubated at 28 °C and 180 rpm for 8 days. All cultivations were made in three biological replicates.

In order to determine the dry cell weight (DCW), 2 mL of the culture was taken from the flasks, washed, and lyophilized in a pre-weighed tube. The differences in mass corresponded to the mg of cells in 2 mL of culture.

### 2.3. Lipid Extraction and Analysis by GC-FID

Lipids were extracted from freeze-dried cells (around 20 mg) by transmethylation described by Browse et al. [18]. The methylated FA (FAMEs) were then analyzed using gas chromatography equipped with a flame ionization detector (GC-FID, Varian 3900, Walnut Creek, CA, USA) and a Varian FactorFour vf-23 ms column where the bleed specification at 260 °C is 3 pA (30 m, 0.25 mm, 0.25 μm).

The FAMEs were identified via comparisons with commercial standards (FAME32, Supelco, Bellefonte, PA, USA) and quantified using the internal standard, 100 μg of commercial dodecanoic acid (Sigma-Aldrich, St. Louis, MO, USA). Commercial standards of OCFAs (Odd Carbon Straight Chains Kit containing 9 FAs, OC9, Supelco, Bellefonte, PA, USA,) were converted into their FAMEs using the same method employed with the yeast samples. They were then analyzed by GC to identify the OCFAs from the yeast samples. For each data point, we used three biological replicates and calculated average and standard deviation values.

### 2.4. Lipid Extraction and Analysis by LC-MS and LC-Corona-CAD^®^

Lipid extraction was carried out by a modified Folch’s method [29] from three independent cultures of *Y. lipolytica* strains JMY7775 and JMY7780. A 4.5 mL volume of chloroform/methanol (1:2) was added to 10 mg of lyophilized *Y. lipolytica* strains and vortexed for 30 s. The mixture was allowed to stay at ambient temperature for 1 h before addition of 1.25 mL of water and vortexing for 30 s. The phase separation was achieved by centrifugation (1000× *g* for 10 min). The (lower) organic phase was collected, and the (upper) aqueous phase was re-extracted by adding 2 mL of chloroform/methanol (85:15). The two organic phases were pooled and evaporated under a stream of nitrogen at room temperature. The dry residue was dissolved in 900 µL of isooctane/chloroform (4:1) before analysis.

The chromatographic conditions of the lipid class analysis have been described previously [30]. The separation was performed with an Inertsil Silica (150 mm × 2.1 mm I.D, 5 mm) column (GL Sciences Inc., Tokyo, Japan) fitted to a Dionex U-3000 RSLC (Thermo Fisher Scientific, Waltham, MA, USA) liquid chromatograph. A quaternary solvent gradient was used to elute all the lipid classes present in the sample by increasing the order of polarity, with slight modifications in the solvent program as presented in [27], where two isocratic steps were introduced to improve PLs separation. The column temperature was set at 25 °C (instead of 40 °C) for the same purpose. The flow rate was set at 0.8 mL/min.

Lipid class identification was verified by coupling chromatographic separation to mass spectrometry. MS analyses were performed with an LTQ-Orbitrap Velos Pro (Thermo Fisher Scientific, Waltham, MA, USA) equipped with an APCI ion source piloted by the Xcalibur software. The MS^2^ and MS^3^ spectra were obtained in data-dependent acquisition (DDA) mode as described previously [30]. The injected volume was increased to 10 µL (instead of 2 µL) to improve the detection.

The TAG, DAG, GlcADG, and PL FAs distribution is determined with the method described in [27,28]. Briefly, the % FAs are obtained by direct measurement of the % intensities of ions B ([MAG+H-H2O]^+^) for each lipid class molecular species distribution analyzed. In order to facilitate the identification, the *m/z* were compared to the database LIPID MAPS^®^. MS2 spectra allowed distinguishing isomers.

Lipid profiles were compared by coupling the chromatographic separation to a Corona-CAD^®^ detector (ESA, Chelmsford, MA, USA) [30]. An injected volume of 2 µL was used for these analyses. The signal was acquired with a Chromeleon data station (Thermo Fisher Scientific, Villebon-sur-Yvette, France). Corona-CAD^®^ is a universal detector used for liquid chromatography and described in [31]. The differences in the composition of the lipid classes in the samples are expressed as peak areas.

## 3. Results

### 3.1. Metabolic Pathways for Lipid Synthesis and Lipid Readjustments

Pathways for lipid synthesis and degradation are well explored in *Y. lipolytica* as a model oleaginous yeast so far [4,32]. The synthesis of phospholipids in *Y. lipolytica* is identified based on the pathway of *Saccharomyces cerevisiae* [33].

De novo lipid synthesis starts from acetyl-CoA and malonyl-CoA building blocks. Fatty acid synthase enzymatic complex (FAS) produces an acyl-CoA pool using acetyl-CoA as an initiation molecule and malonyl-CoA as an elongation unit. The released acyl-CoAs go through further modification, elongation, and/or desaturation, resulting in various fatty acids with different chain lengths and the degree of saturation. These acyl-CoA pools are condensed with glycerol-3-phosphate to generate lysophosphatidic acid (LPA), then phosphatidic acid (PA), diacylglycerol (DAG), and finally triacylglycerol (TAG) via the Kennedy pathway. TAG is then incorporated into the lipid body (LB) specialized compartment for neutral lipid storage.

**Figure 1:**
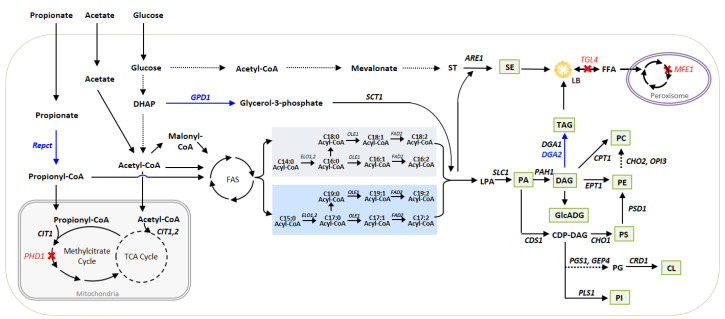
The engineering strategy used in this study to improve the accumulation of odd-chain fatty acids are described in the pathway. The overexpressed genes in the *Y. lipolytica* strain used in this study are written in blue, and corresponding paths are depicted with blue arrows. The deleted genes are written in red, and corresponding paths are depicted with red cross. The lipid class detected in this study are written in green box. The multiple steps in the pathway are depicted as dashed line. Even-chain acyl-CoA pools are in the grey box, and odd-chain acyl-CoA pools are in the blue box. SE—sterol ester; ST—sterol; LPA—lysophosphatidic acid; PA—phosphatidic acid; DAG—diacylglycerol; TAG—triacylglycerol; GlcADG—1,2-diacyl-3-O-α-glucuronosylglycerol; CDP-DAG—cytidine diphosphate-diacylglycerol; PC—phosphatidylcholine; PE—phosphatidylethanolamine; PS—phosphatidylserine; PG—phosphatidylglycerol; CL—cardiolipin; PI—phosphatidylinositol; LB—lipid body; FFA—free fatty acid.

Glycerophospholipids known to compose the plasma membrane have different classes depending on their head group. Each class is synthesized through a different pathway, as depicted in Figure 1. Phosphatidylserine (PS), phosphatidylethanolamine (PE), and phosphatidylcholine (PC) are synthesized through the CDP-DAG pathway. PC has an alternative pathway from DAG, too. CDP-DAG is also a precursor of phosphatidylinositol (PI) and cardiolipin (CL), as well as the products of the CDP-DAG pathway.

Sterol (ST) is synthesized from acetyl-CoA through the mevalonate pathway, then converted to sterol ester (SE), which is known as a storage form of lipids in the cell.

The fatty acids stored in the lipid body can be mobilized and degraded through the β-oxidation pathway, resulting in the release of acetyl-CoA (relevant pathways and genes were well explained in the review [4,32]).

### 3.2. Odd-Chain Fatty Acid Production by Obese L and Obese LP Strains

**Figure 2:**
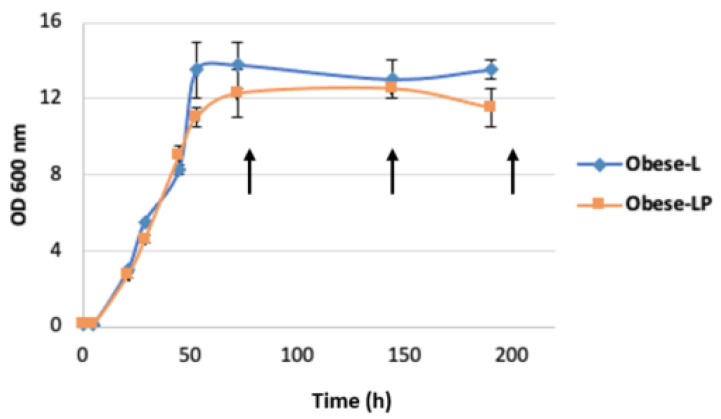
Growth and time-point sampling for lipidomic analysis. Two strains obese L (JMY7775) and obese LP (JMY7780) were grown in YNBDPA media at 28 °C. Samples for lipidomic analysis were taken at 72 h, 144 h, and 216 h (arrow). The corresponding samples were named L-72, L-144, L-216 and LP-72, LP-144, LP-216, respectively. Growth A600 is adapted from Park et al., 2021.

In this study, two *Y. lipolytica* strains (obese L and obese LP) engineered to accumulate a high amount of lipids, mostly TAG, were used. The metabolic modification includes the deletion of *MFE1* and *TGL4* to inhibit TAG remobilization and degradation, the overexpression of *GPD1* and *DGA2* to push and pull TAG biosynthesis, and the overexpression of *LPD1* to enhance the storage of TAG (obese L strain). The OCFA-producing strain (obese LP strain) has a unique modification to improve OCFA synthesis, the introduction of the *PCT* from *Ralstonia eutropha* to boost the propionyl-CoA pool [1]. In order to improve OCFA synthesis, the condition of cultivation, especially the combination of carbon sources and the C/N ratio, was optimized in the same study. Thus, obese L and obese LP strains were cultivated with a substrate combination of 2% (*w*/*v*) glucose, 0.5% (*w*/*v*) propionate, and 1% (*w*/*v*) acetate and the ratio C/N = 45 for the analysis of lipid readjustment in this study. Samples for lipid analysis and lipidomic analysis were harvested at 72 h, 144 h, and 216 h (Figure 2). First, to verify biomass and lipid content, samples from 216 h were analyzed by GC-FID as described previously [1]. Results showed that the major FA produced from the control strain (Obese L) was oleic acid (C18:1), while the obese LP strain produced mostly heptadecenoic acid (C17:1), which is consistent with the previous study. The ratio of OCFA to total lipid was 5.07% in the obese L strain, while more than 50% of lipids were OCFA in the obese LP strain, as described in Table 1. Around 35% of lipids were heptadecenoic acid (C17:1), the major OCFA, in the obese LP strain. There was no significant difference in substrate utilization between the two strains, Figure S1).

### 3.3. LC-Corona-CAD^®^ and LC-MS Analyses

**Figure 3:**
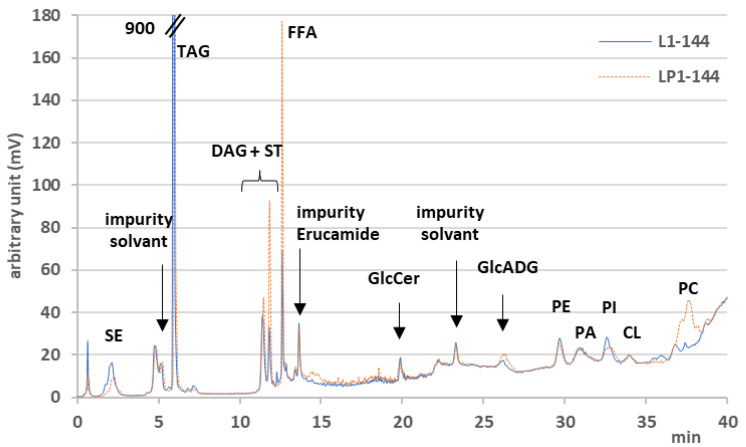
LC-Corona-CAD^®^ lipid profiles from obese L1-144 and obese LP1-144. SE—sterol esters; TAG—triacylglycerols; DAG—diacylglycerols; ST—sterols, FFA—free fatty acids; GlcCer—glucosylceramides; GlcADG—glycosyldiacylglycerols; PE—phosphatidylethanolamines; PA—phosphatidic acids; PI—phosphatidylinositols; CL—cardiolipins; PC—phosphatidylcholines.

The lipid samples from the obese L and obese LP strains, at the three time points, were analyzed by LC-Corona-CAD^®^ a in triplicates and were named one to three. Results from sample one, collected at 144 h of cultivation, are shown in Figure 3. Twelve different lipid classes were separated and identified using this chromatographic method. Results are from three independent extractions. Chromatogram corresponds to sample one at 144 h for the strain obese L (obese L1-144) and obese LP (obese LP1-144). TAG is the primary lipid class in *Y. lipolytica*. In Figure 3, the TAG peak (measured at 900 mV) is truncated to allow the observation of other lipid classes, which are SE, DAG, FFA, GlcCer, GlcADG, PE, PA, PI, CL, and PC. DAGs are eluted as a double peak (1,3 DAG and 1,2 DAG); however, the literature describes changes in stereochemistry during analysis that do not reflect the true stereochemistry in the sample [34]. For this reason, the data from the two peaks were summed, and the stereochemistry was not taken into account. The LC-MS data allowed identifying a small amount of ST co-eluted with the first DAG peak. Peaks related to impurities present in the solvents or plastic (Erucamide) [35] are also observed, with no impact on the analysis.

In the following figures, the ordering of the lipid classes corresponds to the order of elution. FAs and lipid molecular species will be presented in increasing molecular masses.

#### 3.3.1. Storage Lipid Profiles during Odd-Chain Fatty Acid Production

**Figure 4:**
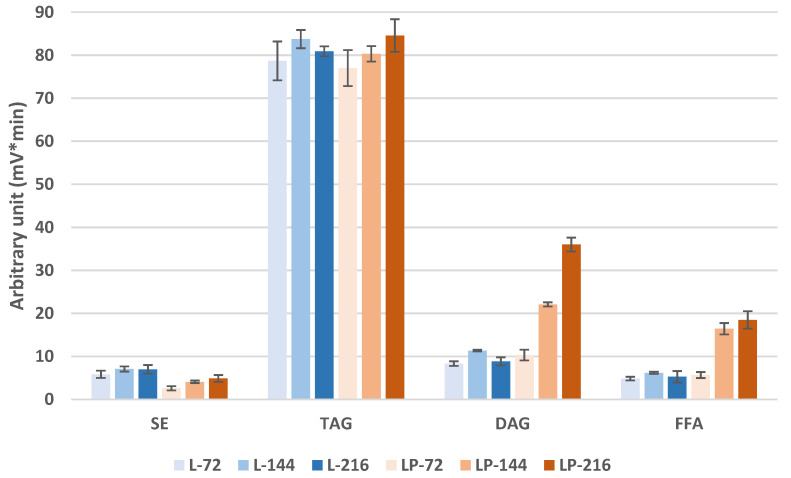
Comparison of the storage lipid content in the obese L and obese LP over time. SE—sterol esters; TAG—triacylglycerols; DAG—diacylglycerols; FFA—free fatty acids. Mean values are displayed (*n* = 3, ± SD).

Figure 4 represents the peak areas of the storage lipid classes as obtained in LC-Corona-CAD^®^.

TAG amounts are comparable between the two strains and do not vary significantly over time, as described in Figure 4. Similarly, the amounts of SEs, DAGs, and FFAs were similarly maintained over the cultivation time in the obese L strain. On the contrary, the OCFA-producing strain (obese LP), the odd-chain-producing strain, presents a time-dependent increase, in particular for DAGs and FFAs. SE levels are lower in obese LP compared to the control, suggesting limitation in the Kennedy pathway. Alternatively, this increase in DAG and FFA may result from the induction of the expression of lipase genes, such as the *LIP2* gene coding for the main extracellular lipase Lip2p, or the expression of intracellular lipases coding genes such as *LIP1*, *LIP3,* and *LIP5*, or the induction of the TAG remobilization pathway by increasing the expression of the genes coding for the intracellular TAG-lipases Tgl3p and/or Tgl4p, involved in TAG remobilization.

**Figure 5:**
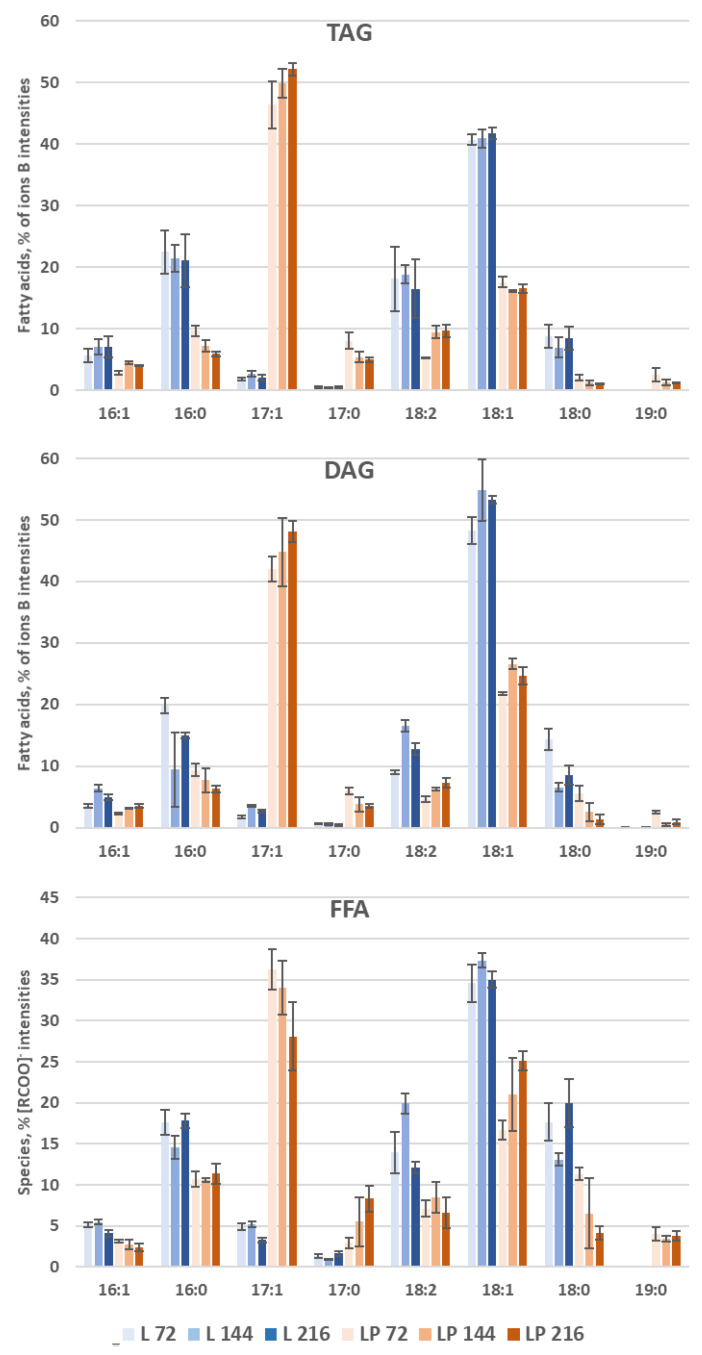
Comparison of the storage lipid composition in the obese L and obese LP over time. Lipid profiles in % FA per classes for TAG and DAG and % [RCOO]^−^ Intensities for FFA. Mean values are displayed (*n* = 3, ±SD). Lipid species below 3% are not indicated.

LC-MS analyses allow access to the FA distribution of most lipid classes. The distribution of FAs of TAGs and DAGs is obtained from the relative intensities of ions [MG+H-H_2_O]^+^ [27,28]. In the case of free FFAs, where this approach cannot be implemented, the relative intensities of the [RCOO]^-^ ions are presented. The FA distribution is presented in Figure 5.

Obese LP produces significantly more OCFAs than obese L for these three reserve lipids. The majority of FA is C17:1 in obese LP and C18:1 in obese L. In general, all even FAs decrease in favor of mainly C17:1 in obese LP. Moreover, in obese LP, TAGs, and DAGs, unsaturated FAs (C17:1 and C18:2) increase while saturated FAs (C16:0, C17:0, C18:0, and C19:0) decrease.

SE mass spectra obtained for obese L and obese LP at 144 h in positive and negative ionization modes are presented in Figure S2. In positive mode, SE directly loses their FA, and the majority ion corresponds to [M-FA+H-H_2_O]^+^ [36]. This ion gives information on the sterol nucleus but does not allow for formally identifying the sterol among the numerous existing isomers. The nature of the sterols nuclei naturally present in *Y. lipolytica* SEs has already been investigated in the literature [37,38,39]. The identification of the sterols nuclei is presented in Table S1, and the distribution of [M-FA+H-H_2_O]^+^ ions are presented in Figure S3. The most abundant SE contains ergosta 5,7 dienol, corresponding to ergosterol with two π-bonds and an additional methyl group on the B-ring and acyl chain. High intensities corresponding to ergosterol and episterol are also measured. The distribution of sterol nuclei in SE is slightly different depending on the strain. Obese LP has a higher proportion of ergosta 5,7 dienol and ergosterol and a lower proportion of episterol and lanosterol than obese L.

In negative mode, the fragment ions [RCOO]^-^ of the full scan mass spectra may, to some extent, indicate the distribution of esterified FAs on the SEs. Obese L1-144 and obese LP1-144 spectra are presented in Figure S4. These spectra show a similarity in the distribution of FAs in this class and those of other storage lipids. 

Under our analytical conditions, it is not possible to formally identify the molecular species of SE, TAG, and DAG.

#### 3.3.2. Membrane Lipid Profiles during Odd-Chain Fatty Acid Production

**Figure 6:**
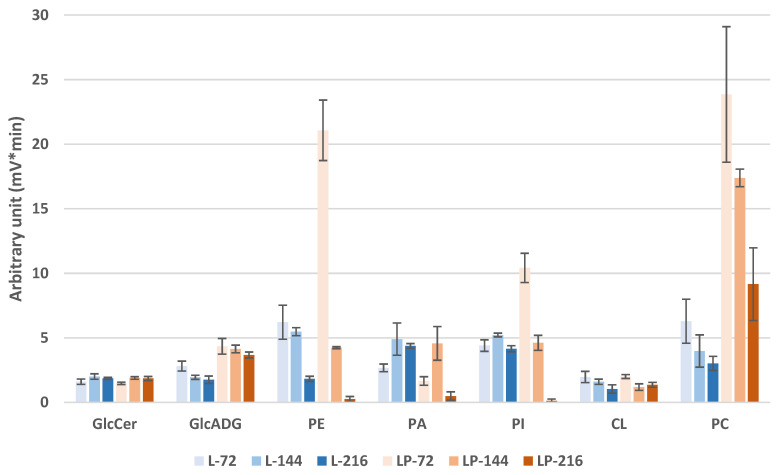
Comparison of membrane lipid composition in the obese L and obese LP over time. Error bars represent the standard deviation of triplicates. Mean values are displayed (*n* = 3, ±SD). Lipid classes below 3% are not indicated.

Figure 6 represents the peak areas obtained in LC-Corona-CAD^®^ of the membrane lipids of the different samples. ST are omitted in this figure since their abundance is very low, and the ST peak is partially coeluted with DAGs. 

At 72 h, obese LP contains much higher PE, PI, and PC amounts than obese L. These amounts decrease drastically at 144 h and 216 h. At 216 h, PE and PI have almost disappeared from the LC-Corona-CAD^®^ profiles. However, the PC remains at a high level compared to obese L.

In obese L, PE and PC decrease less significantly, and PI remains stable over time. PA is the only PL whose amount increases between 72 and 144 h in both strains. CL is present at the same amount in both strains and a decrease with time is visible in both strains. 

The amounts of GlcCer are stable over time and comparable between the two strains. The amounts of GlcADG are higher in obese LP. A slight decrease is observed in both strains.

**Figure 7:**
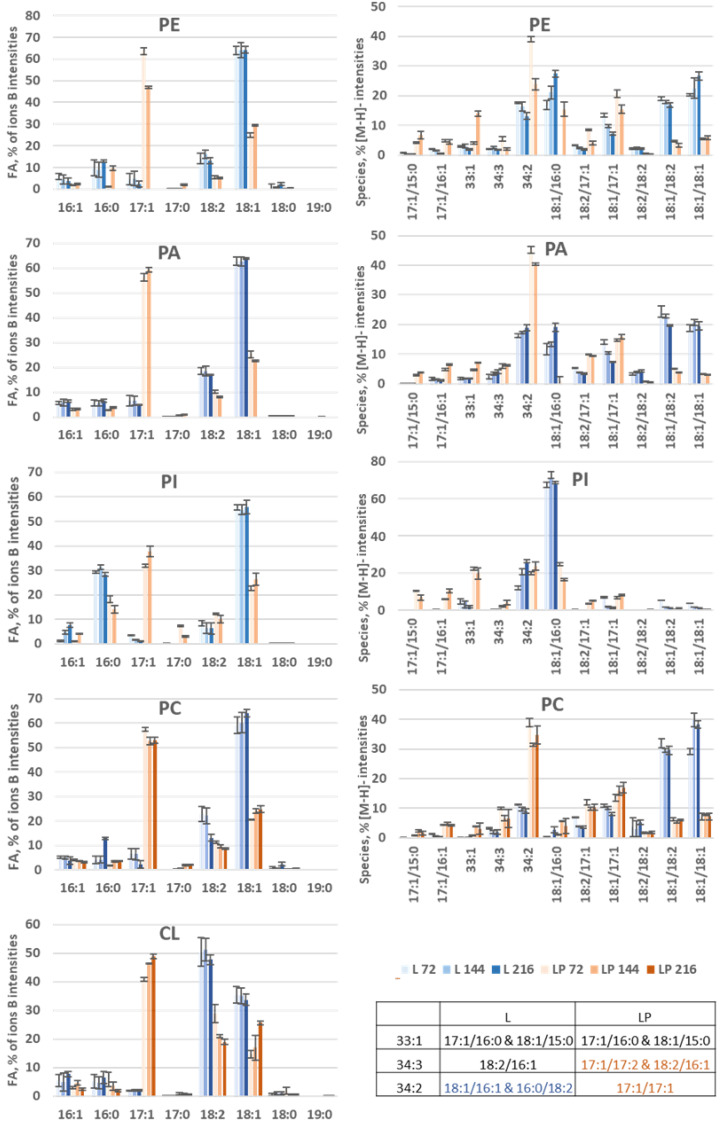
Comparison of the FA profiles in PL classes during OCFA production in the obese L and obese LP over time. On the left side, histograms represent the FA profiles expressed as % of total fatty acids. On the right side, histograms represent the % intensity of PLs molecular species (calculated from their [M-H]^−^). Species noted 33:1, 34:3 and 34:2 correspond to isobaric species differing according to strains. The corresponding molecular species are indicated in the table.

The distribution of FAs in PLs determined by LC-MS [27,28] is presented in Figure 7, left side. At 216 h, the amounts of PE, PA, and PI in obese LP are too low to be exploited by LC-MS. Obese LP produces significantly more OCFAs than obese L in the membranes. The major OCFA is consistently C17:1 in obese LP and C18:1 in obese L, except for CL (mainly C18:2). C17:0 is predominantly detected in PI (3% and 7%). C19:0 represents less than 0.1% of the FAs in PLs, whereas it could represent up to 3% of the FAs in storage lipids. In general, all even FAs decrease in favor of mainly C17:1 in obese LP. The distribution in FAs is comparable between PE, PA, and PC. PI differentiates itself by a higher % of C16:0 and CL by a higher percentage of C18:2. CL is mainly localized in mitochondria and predominantly contains C18:2 FAs [40]. 

#### 3.3.3. Species Distribution

- PLs

The molecular species distribution, shown on the right side of Figure 7, was determined from the intensity of [M-H]^−^ ions for all PLs and, specifically for PC, [M°]^−^. FAs identification was performed from the MS² mass spectra of the corresponding ions. An example of a full scan and MS² mass spectrum of the most abundant ion for obese L and obese LP at 144 h of each PL class is shown in Figure S5–S12. Table S2–S5 present the identification of the [M-H]^−^ or [M°]^−^ of PLs. As the ionization of PLs is dependent on the nature of the FAs and the physiochemical environment [41], the concordance of these data with those obtained by ions B [27,28] was investigated. Therefore, the FAs distribution was recalculated from the results issued from the distribution of molecular species, and the results are presented in Figure S13. The distributions of FAs calculated in this way and those obtained by B ions show the same trends for all PLs. An overestimation is observed for the C17:1 of PI, which is explained by the presence of isobaric molecular species of PI (17:1/17:1), not taken into account in the calculation. This overall agreement of the two methods provides reasonable confidence in the molecular species distribution data. CL is the only PL studied containing four FAs; the molecular species representation for this class is presented differently in the following.

The species noted in 33:1, 34:3, and 34:2 correspond to isobar molecular species; the correspondence is given in the box of Figure 7. All molecular species in obese L containing C17:1 are more abundant in obese LP (at the detriment of the other species). The 17:1/17:1, almost non-existent in obese L, represents the most abundant molecular species in obese LP except in the PI, where it is at the same level as 18:1/16:0 and 17:1/16:0. There are no single predominant molecular species in obese L, except in the PI with PI (18:1/16:0). On the other hand, although the distribution of FAs is similar for PE, PA, and PC, the distribution in terms of molecular species is not uniform. In obese L, PC is distinguished by two majority species, PC (18:1/18:2) and PC (18:1/18:1), whereas in PE and PA, there are four majority species. In addition, PC has a very low percentage of 18:1/16:0 compared to other PLs. In obese LP, the distribution of species in PA and PC are close. The majority species is the (17:1/17:1), whereas, in the PE, four molecular species are in the majority representing about 20%.

- CL

For CL, a full scan mass spectrum is presented for each strain in Figure S14. Molecular species are identified by the number of carbons shown at the bottom of the figure and by the number of unsaturations shown at the top of the ion intensities. Examples of the most likely molecular species (based on the distribution of the previously determined FAs) are given as a guide. Fragmentation could not be exploited due to the low intensities of [M-H]^-^ ions. The spectra show that obese L contains predominantly two molecular species, C72:7 and C72:6 (probably corresponding to CL (18:2/18:2/18:2/18:1), and CL (18:2/18:2/18:1/18:1). Obese LP has a wider distribution with shorter chains due to the abundance of C17:1.

- GlcADG

**Figure 8:**
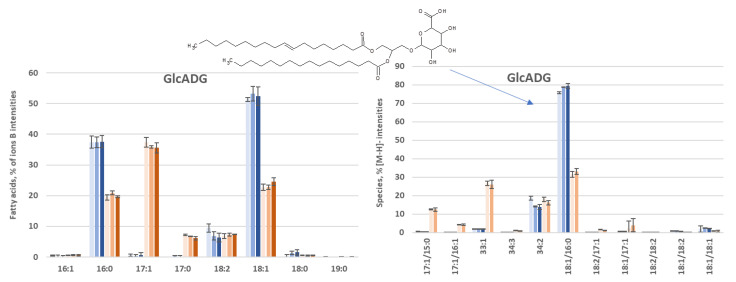
Comparison of %FA profiles in the GlcADG during OCFA production in the obese L (Blue) and obese LP (orange) over time (72, 144 and 216 h).

The FA and molecular species distribution of GlcADG are presented in Figure 8. The distributions are very similar to those found for PI. This is probably related to their chemical structures, which are also very close. The mass spectra are presented in Figure S15, and the MS² of the majority [M-H]^-^ ion is shown in Figure S16.

- GlcCer

Since GlcCer does not contain a glycerol backbone but a sphingoid base, the FAs distribution cannot be estimated from the ion B measurement. The obese L and obese LP mass spectra at 144 h in negative and positive ionization mode are shown in Figure S17. GlcCer ionizes as [M-H]^-^ and [M+H-H2O]^+^, respectively. According to the LIPID MAPS^®^ MS Data Bulk Search tool, the [M+H-H2O]^+^ ion at 710.552 *m/z*, which is predominant in both strains, is consistent with a HexCer 35:2;O_3_. This structure is in agreement with the observations of Bal and coworkers [42], who reported that the sphingolipids synthesized by *Y. lipolytica* are GlcCer with a long chain base C18, desaturated at C4 and C8 positions and methylated at C9, and an amide-bonded C16 FA hydroxylated at the C2 position. 

In obese LP, the [M+H-H2O]^+^ ion at 724.568 *m/z* is also very abundant (60% of the intensity of the major ion). By analogy, we hypothesize that its structure contains a C17 FA instead of C16.

- ST

LC-MS allows selectively extracting the spectrum of ST from their coelution with DAGs. For STs, a mass spectrum for obese L and LP at 144 h in positive mode (in the range [360–400]) is shown in Figure S18. The only ST in both strains is ergosterol ([M+H-H_2_O]^+^ ion at *m/z* 379.335. The intensities are of the same order of magnitude in both strains. The same observation was made for the other time points (data not shown). In contrast to SE, which contains a diversity of sterol nuclei, free STs are exclusively composed of ergosterol.

## 4. Discussion

*Y. lipolytica* is widespread in nature. This yeast has often been found to inhabit dairy products, cheese, contaminated milk, fermented vegetables, poultry, sausages and meat products, and marine ecosystems, including high saline waters, as well as in environments rich in hydrophobic substrates [43,44].

This yeast was shown to be well adapted to efficiently utilize hydrophobic substrates such as n-alkanes, fatty acids, fats, and oils [45,46,47], which correlate with its ecologic niches such as sewage, industrial wastewaters, oil-polluted soil, and seawater [44].

This non-conventional strictly aerobic ascomycetous yeast, generally regarded as safe (GRAS) by the American Food and Drug Administration (FDA), has been exploited in several biotechnological, environmental, and industrial applications. Some examples include heterologous hosts for producing pharmaceutically and industrially relevant proteins, enzymes, organic acids, biofuels, and bioremediation of industrial and environmental waste.

The capacity of *Y. lipolytica* to degrade very efficiently hydrophobic substrates, for which it has a specific metabolic pathway, made this yeast a promising chassis as a cell factory for producing lipid and lipid derivatives.

The oleaginous yeast *Y. lipolytica* is a particularly attractive platform for the sustainable industrial production of lipid-derived fuels and chemicals [3] and the production of usual and unusual lipids [4].

Over the past several years, great progress has been made to improve lipid and lipid-derivatives production by *Y. lipolytica* [48,49,50]. Different strategies have been used for increasing lipid accumulation and recovery from *Y. lipolytica* [51,52].

Several engineered strains have been generated to over-accumulate specific fatty acids, such as the high oleic acid producing strains, where oleic acid represents more than 90% of the fatty acids [53], 6.5% of conjugated linoleic acid [54], Cyclopropane fatty acids reach 22% [55], 10-methyl branched fatty acids represent 37% [56] and odd chain fatty acids content was improved from 46.8% up to 62% [1,5].

The results of our study show about a 22% lipid content in our condition for both strains (obese and OCFA producing strains). Similar lipid content was previously reported by Sekova and coworkers [22] and in our previous reports [1]. Additional genetic modifications could be introduced to further improve the lipid content in odd-chain-producing strains, as those described in the recent review by El kantar and Koubaa [52].

Few lipidome analyses were previously reported; Hein and Hayen reported the comparison of lipidome in different yeast [21]. Furthermore, Sekova and coworkers [22] reported a lipidome analysis of the *Y. lipolytica* wild-type strain W29 at two temperatures of culture (29 °C and 38 °C) and two pH (5.5 and 9). A high level of DAG was observed at 38 °C (20–30% of storage lipid), which were not present at a lower temperature. A two-fold decrease in the FFA level was reported (from 40% to about 20%). The optimal lipid content of cell dry weight was 23% at pH 5.5, 29 °C, while it represents only 14,4% at pH 9, 38 °C at the expense of a two-fold higher level of cytosol sugar (a mean of 4.4% at 29 °C, up to a mean of 10.4% at 38 °C). Our study shows no changes in TAG level in obese L versus obese LP (Figure 4); however, an increase in DAG and FAA in the obese LP during cultivation suggests a lipid readjustment induced by stress resulting from the OCFA production. Such lipid readjustments were induced by temperature stress but not pH stress [22].

A transitory lipid readjustment during OCFA production was mainly observed in the membrane lipid composition at the PE, PI, and PC levels and was mainly seen at 72 h of culture (Figure 6). In contrast, Sekova and coworkers did not observe PE readjustment but observed an increase in PC at 38 °C [22]. We may hypothesize that such transitory readjustment may occur upon temperature stress if a lipidomic analysis has been performed during the culture time. Alternatively, we could speculate that the temperature stress response may be different from the membrane fatty acid profile response.

This lipidomic study is the first implementation of the FAs distribution assessment by NPLC-HRMS as proposed by [27,28]. According to this method, the FAs distributions of glycero- and glycerophospholipid classes are assessed from their insource fragmentation acquired during the lipid classes chromatographic separation. This information is comparable with the classical methodology, which consists of a TLC separation followed by a GC-FID FAMEs analysis as used in Sekova et al.’s study [22]. This new methodology is simple to implement and much more rapid than the classical one but does not provide information about unsaturation positions. It provides the FAs with relative distribution, the most frequently reported result, but not a quantitation of individual FAs. This rapid method of FAs distribution assessment is advantageous when an important number of samples is to be analyzed. This allowed us to perform a kinetic study at three different times and to evidence the PE readjustment. Furthermore, the lipid extract can be analyzed without pretreatment or sample denaturation. The whole information is accessible, and a non-targeted analysis can be conducted.

Studies using HILIC [38] or RPLC [57] coupled with ESI-MS are comparable to our approach in their implementation but do not allow assessing the FAs distribution of lipid classes. In both HILIC and RPLC studies, GC-MS complementary analyses were conducted to evaluate the total FAs distributions. This total FAs distribution is less informative than the per-class distribution.

As previously indicated, the objective of our lipidomic approach was to evaluate the readjustment of OCFAs in two engineered *Y. lipolytica* strains. For this purpose, an NPLC-Corona-CAD^®^ analysis [30] allowed a detailed analysis of the relative contents of the different lipid classes with a time-dependent over-expression of FFAs, DAGs, and PCs in obese LP compared to obese L. As proposed in two recent studies [27,28], coupling the NPLC method to APCI+-HRMS allows the assessment of the distribution of FAs, namely the different glycero- and glycerophospholipid classes. This approach offers an important degree of detail for each of these lipid classes and allows, if necessary, to compare this distribution to that of the molecular species. Thus, we were able to verify that TAG and DAG had a similar FA distribution with C18:1 as the majority FA in obese L, which C17:1 replaces in obese LP. The observation is similar in the PL classes, although more subtle differences can be noted. Thus, the distribution of FAs in PA, PE, and PC are similar and close to those of TAG and DAG, with here also a predominance of C18:1 in obese L replaced by C17:1 in obese LP. In obese L, C18:1 is distributed in three main species (PC (18:1/16:0), PC (18:1/18:1), and PC (18:1/18:2)) of equal importance, while C17:1 is found overwhelmingly in PC (34:2) in obese LP. CL and PI show differences from the other classes of PLs. In obese L, PI shows a distribution of FAs where C18:1 is in the majority associated with a high proportion of C16:0, which leads to the presence of the very majority species PI (18:1/16:0). In contrast, in obese LP, FA C17:1 alongside C16:0 and C18:1 leads to a complete redistribution of these FAs within the molecular species. Concerning CL, C18:2 is in the majority in obese L next to C18:1, while these two FAs remain present although halved in favor of C17:1 in obese LP. 

CL is known to undergo a remodeling favoring C18:2 incorporation [58]. A high level of C18:2 is correlated with an effective mitochondrial function. CL is primarily located in the inner membrane of mitochondria, notably in the cristae region [59].

When an aberrant distribution of CL FAs is observed, as in Barth’s syndrome [60,61], mitochondrial function is impaired, including energy production and protein transport. In the case of obese LP, the distribution of CL FAs is strongly altered; it is reasonable to assume that this may negatively affect the development of these strains.

Based on the experimental evidence, we conclude that *Y. lipolytica* can use different kinds of lipid readjustment responses for long-term adaptation to unfavorable environmental conditions and upon unusual fatty acids production.

Based on the modification in FA profiles and phospholipid type and profiles, it is worth noticing that lipid readjustments take place rapidly. Accumulation of unusual lipids in *Y. lipolytica* leads to dramatic metabolic readjustments of lipid profiles and crucial changes in the membrane lipids and sterols. 

The flexibility and the readjustments of lipid and phospholipids upon OCFA synthesis demonstrate that *Y. lipolytica* has the capacity to modulate and induce lipid readjustment. This confirms that *Y. lipolytica* has high potential as a workhorse microorganism for producing unusual lipids. 

## Figures and Tables

**Table 1:** Biomass and lipid content from obese L and obese LP strains. The samples harvested at 216 h were used for the analysis of GC-FID.

Samples	DCW (g/L)	Lipid Content % (g/g DCW)	Lipids (g/L)	OCFA /Total Lipids (%)	C17:1 /Total lipids (%)	C17:1 /C18:1 (%)
Obese L	8.48	21.46	1.82	5.07	2.30	5.47
Obese LP	6.07	23.92	1.45	50.86	35.29	197.98

## Data Availability

Mass spectrometry data are available upon request to P. Chaminade.

## Supplementary Materials

The following are available online at https://www.mdpi.com/article/10.3390/biom12081026/s1, Figure S1: Substrates consumption during cultivation. Figure S2: SE mass spectra of obese L and obese LP at 144 h. Figure S3: Distribution of [M-FA+H-H2O]^+^ ions and corresponding stérols in SE fraction. Figure S4: SE mass spectra in obese L and obese LP at 144 h, zoom *m/z* [225–300]. Figure S5: PE mass spectra in obese L and obese LP at 144 h. Figure S6: MS² spectra of ions @714.55 of detected PE. Figure S7: PA mass spectra in obese L and obese LP at 144 h. Figure S8: MS² spectra of ions @671.46 of detected PA in obese L and obese LP at 144 h. Figure S9: PI mass spectra in obese L and obese LP at 144 h. Figure S10: MS² spectra of ions @833.52 of detected PI in obese L and obese LP at 144 h. Figure S11: PC mass spectra in obese L and obese LP at 144 h. Figure S12: MS² spectra of ions @757.50 of detected PC in obese L and obese LP at 144 h. Figure S13: Obese L and obese LP (at 144 h) FA distribution of PLs classes calculated from Ion B intensities (green bars) or from molecular species intensities (yellow bars). Figure S14: CL mass spectra in obese L and obese LP at 144 h. Figure S15: GlcADG mass spectra in obese L and obese LP at 144 h. Figure S16: MS² spectra of ions @769.54 of detected GlcADG in obese L and obese LP at 144 h. Figure S17: GlcCER mass spectra in obese L and obese LP at 144 h. Figure S18: ST Mass spectra of Ergosterol in obese L and obese LP at 144 h. Table S1: Identification of sterol motifs present in SE. Table S2: Identification of molecular species present in PE. Table S3: Identification of molecular species present in PA. Table S4: Identification of molecular species present in PI. Table S5: Identification of molecular species present in PC.

## Abbreviations

ACPT	acyl-ACP thioesterase
AcTAGs	acetylated TAGs
C/N ratio	carbon to nitrogen ratio
CFA	cyclopropanated fatty acid
CL	cardiolipin
CLA	conjugated linoleic acid
DacT	diacylglycerol acetyltransferase
DAG	diacylglycerol
DCW	dry cell weight
DHA	docosahexaenoic acid
DPA	docosapentaenoic acid
ECFA	even-chain fatty acid
EPA	eicosapentaenoic acid
ER	endoplasmic reticulum
FA	fatty acyl
FAEE	fatty acid ethyl ester
FAME	fatty acid methyl ester
FAS	fatty acid synthase
FFA	free fatty acid
GPL	glycerophospholipids
KAS	beta-ketoacyl-ACP synthase
KS	ketoacyl synthase
LA	linoleic acid
LB	lipid body
LCFA	long-chain fatty acid
LPA	lysophosphatidic acid
MAG	monoacylglycerol
MCFA	medium-chain fatty acid
MFE	multi-functional enzyme
MPT	malonyl/palmitoyl transacylase
OCFA	odd chain fatty acids
PA	phosphatidic acid
PC	phosphatidylcholne
PCT	propionyl-CoA transferase
PE	phosphatidylethanolamine
PG	phosphatidylglycerol
PI	phosphatidylinositol
PKT	polyketide synthase
PPP	pentose phosphate pathway
PS	phosphtidylserine
PUFA	polyunsaturated fatty acid
RA	ricinoleic acid
SCFA SE ST	short-chain fatty acid sterol ester sterol
TAG	triacylglycerol
TALEN	transcription activator-like effector nuclease
VLCFA	very long-chain fatty acid
WT	wild-type
