# Supplementary material for: Lipid Readjustment in Yarrowia lipolytica Odd-Chain Fatty Acids Producing Strains
Source: Biomolecules. 2022 Jul 25;12(8):1026. doi: 10.3390/biom12081026 (PMC9394261; doi:10.3390/biom12081026)
Supplement: Supplementary file 1 [file biomolecules-12-01026-s001.zip › biomolecules-1804791-supplementary.pdf]

biomolecules

MDPI

Article

Lipid Readjustment in *Yarrowia lipolytica* Odd-Chain Fatty Acids Producing Strains

Sonia Abreu[1,†], Young-Kyoung Park[2,†], Camilla Pires de Souza[2], Lea Vidal[2], Pierre Chaminade[1,*] and Jean-Marc Nicaud[2,*]

[1] Université Paris-Saclay, Lipides: Systèmes Analytiques et Biologiques, 92290, Chatenay-Malabry, France; sonia.abreu@universite-paris-saclay.fr (S.A.); pierre.chaminade@universite-paris-saclay.fr (P.C.)
[2] Micalis Institute, INRAE, AgroParisTech, Université Paris-Saclay, 78350 Jouy-en-Josas, France; yk16.park@gmail.com (Y.K.P.); camilla.pires-de-souza@inrae.fr (C.P.); lea.vidal@inrae.fr (L.V.); jean-marc.nicaud@inrae.fr (J.M.N.)
* Correspondence: jean-marc.nicaud@inrae.fr; pierre.chaminade@universite-paris-saclay.fr
† These authors equally contributed to the study and should be considered as co-first authors.

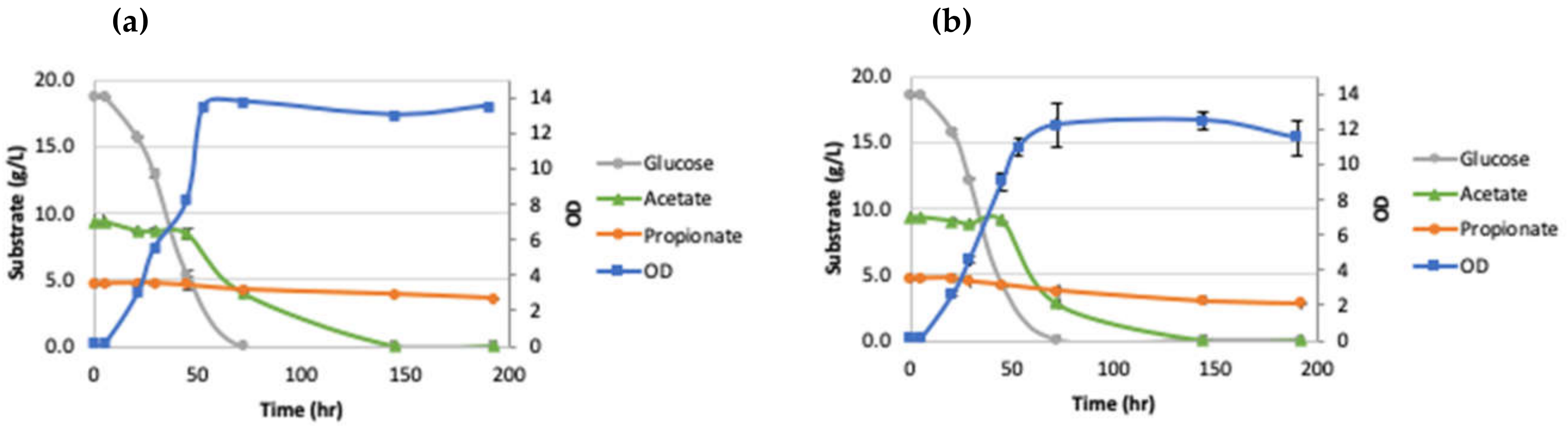

Figure S1. The consumption of substrates during the cultivation. (a) control, obese-L strain, (b) OCFA-producer, obese-LP strain. Averages and standard deviations were obtained from two replicate experiments. This data was published in Park et al. 2021.

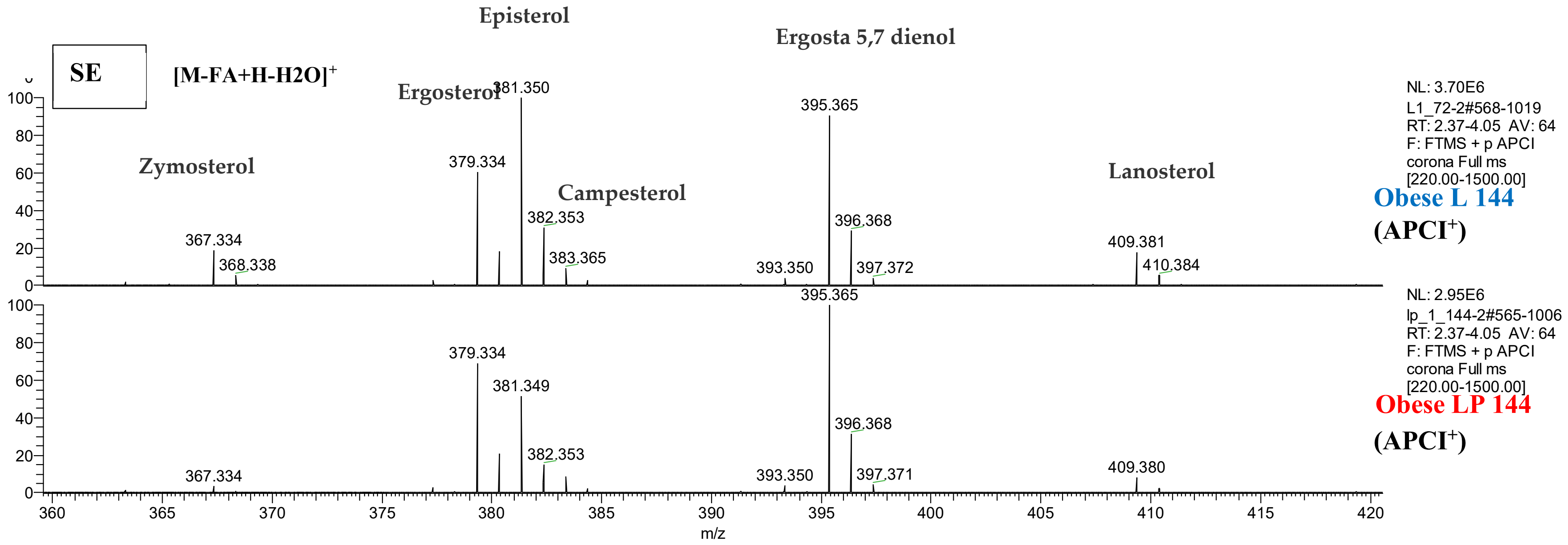

Figure S2. SE mass spectra of obese L and obese LP at 144 hours. The obseved ions correspond to [M-FA+H-H2O]$^{+}$.

Table S1. Identification of sterol nuclei present in SE. The m/z observed were imput in the Lipid Maps database (column 1). The corresponding matched mass are indicated in column 2. The delta mass, the name, the formula and the corresponding Ion are indicated in column 4 to 5, respectively. In column 7, the putative corresponding sterols according to the Lipid Maps and the identified Yarrowia lipolytica sterols by Walker et al. (2019) are indicated.

Input Mass	Matched Mass	Delta	Name	Formula	Ion	Sterols (putative identity)
367.334	367.3359	0.0019	ST 27:2;O	C27H44O	[M+H-H2O]+	Zymosterol
377.319	377.3203	0.0013	ST 28:4;O	C28H42O	[M+H-H2O]+	Ergosta-5,7,22E,24(28)-tetraenol
379.335	379.3359	0.0009	ST 28:3;O	C28H44O	[M+H-H2O]+	Ergosterol
381.35	381.3516	0.0016	ST 28:2;O	C28H46O	[M+H-H2O]+	Episterol / Fecosterol
383.365	383.3672	0.0022	ST 28:1;O	C28H48O	[M+H-H2O]+	Campesterol
393.35	393.3516	0.0016	ST 29:3;O	C29H46O	[M+H-H2O]+	
395.365	395.3672	0.0022	ST 29:2;O	C29H48O	[M+H-H2O]+	Ergosta 5,7 dienol
409.381	409.3829	0.0019	ST 30:2;O	C30H50O	[M+H-H2O]+	Lanosterol

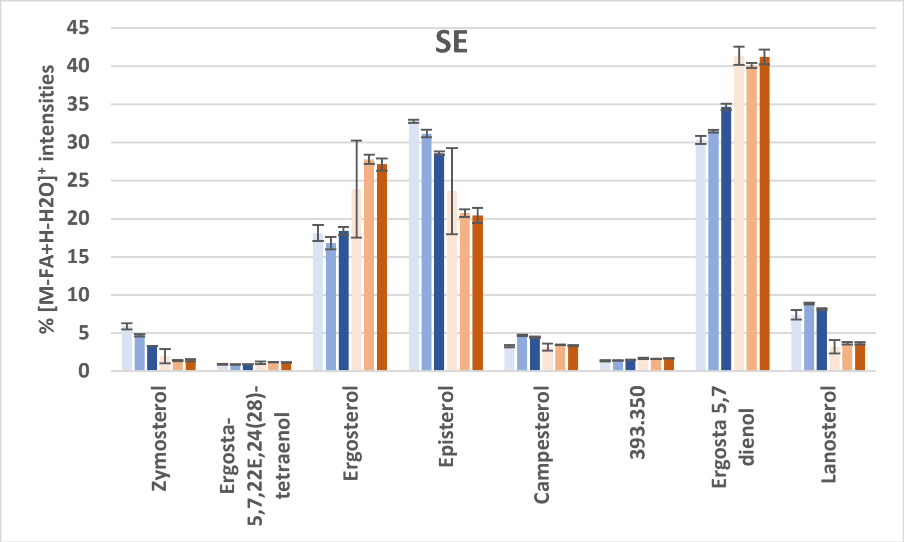

Figure S3. Distribution of [M-FA+H-H2O]$^+$ ions and corresponding sterols in SE fraction in the obese L (blue) and obese LP (orange) at 72, 144 and 216 hours.

.

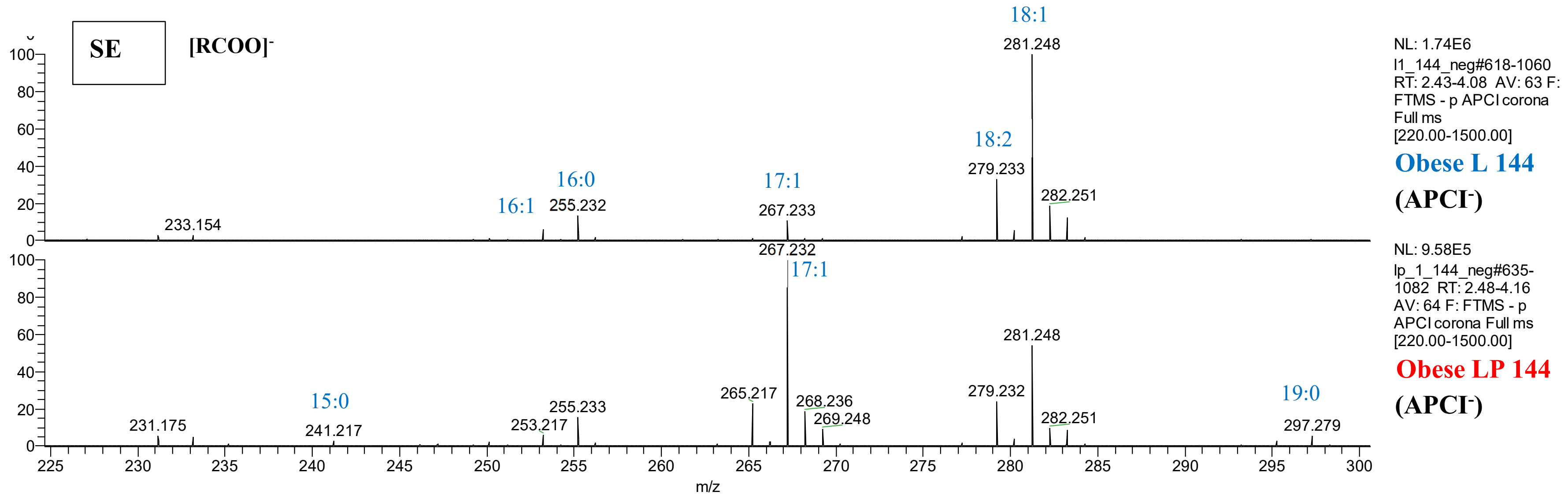

69
70

Figure S4. SE mass spectra in obese L and obese LP at 144 hours, zoomed in the m/z [225-300] region. The observed [RCOO]-, allowing to determine the esterified FA in the sterols. 71 72

73

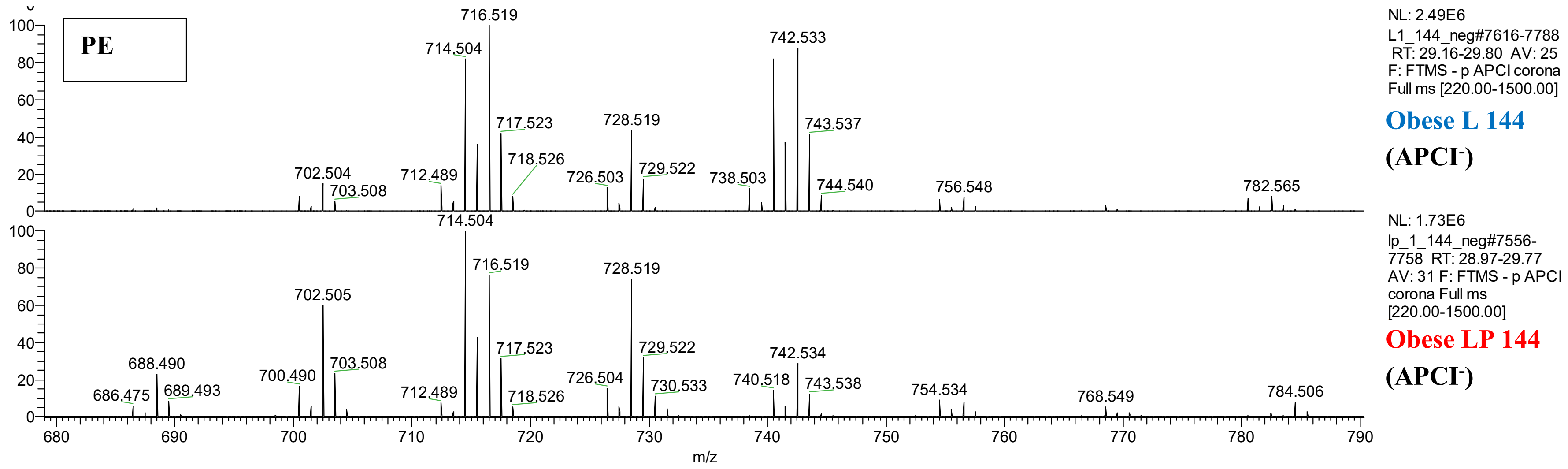

Figure S5. PE mass spectra in obese L and obese LP at 144 hours.

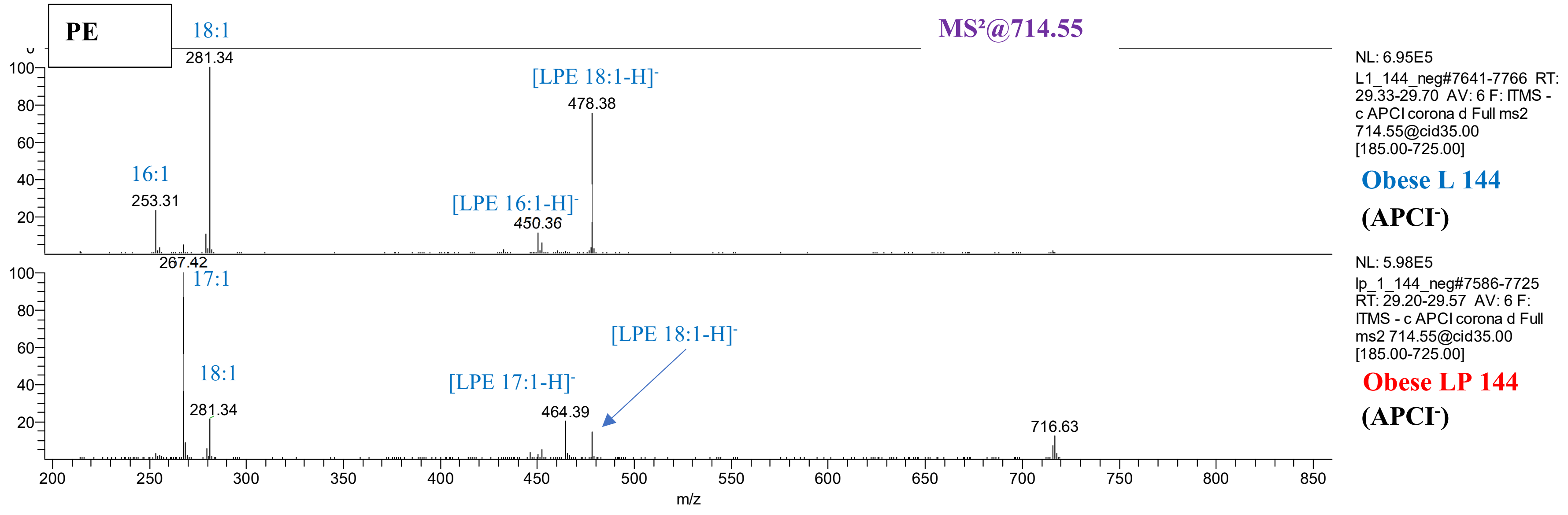

Figure S6. MS² spectra of ions @714.55 of PE in obese L and obese LP at 144 hours.

Table S2. Identification of molecular species present in PE. The m/z observed were imput in the Lipid Maps database (column 1). The corresponding matched mass are indicated in column 2. The delta mass, the name, the formula and the corresponding Ion are indicated in column 3 to 6, respectively. In column 7 and 8, the main molecular species identified according to their name and MS^2 are indicated for obese L and obese LP, respectively.

Input Mass	Matched Mass	Delta	Name	Formula	Ion	Obese L	Obese LP
686.4746	686.4766	0.002	PE 32:2	C37H70NO8P	$[M-H]^-$		17:1/15:1
688.4897	688.4923	0.0026	PE 32:1	C37H72NO8P	$[M-H]^-$		17:1/15:0
700.4898	700.4923	0.0025	PE 33:2	C38H72NO8P	$[M-H]^-$	17:1/16:1	17:1/16:1 & 18:1-15:1
702.5048	702.5079	0.0031	PE 33:1	C38H74NO8P	$[M-H]^-$	17:1/16:0 & 18:1/15:0	17:1/16:0 & 18:1-15:0
712.4894	712.4923	0.0029	PE 34:3	C39H72NO8P	$[M-H]^-$	18:2/16:1	17:1-17:2
714.5043	714.5079	0.0036	PE 34:2	C39H74NO8P	$[M-H]^-$	18:1/16:1 &	17:1/17:1
716.5188	716.5236	0.0048	PE 34:1	C39H76NO8P	$[M-H]^-$	18:1/16:0	17:1/17:0 & 18:1/16:0
726.5035	726.5079	0.0044	PE 35:3	C40H74NO8P	$[M-H]^-$	18:2/17:1	18:2/17:1 & 18:1/17:2
728.5188	728.5236	0.0048	PE 35:2	C40H76NO8P	$[M-H]^-$	18:1/17:1	18:1/17:1
730.5331	730.5392	0.0061	PE 35:1	C40H78NO8P	$[M-H]^-$	18:2-18:2	18:1/17:0 & 18:0/17:1
740.5184	740.5236	0.0052	PE 36:3	C41H76NO8P	$[M-H]^-$	18:1/18:2	18:1/18:2
742.5342	742.5392	0.005	PE 36:2	C41H78NO8P	$[M-H]^-$	18:1-18:1	18:1/18:1

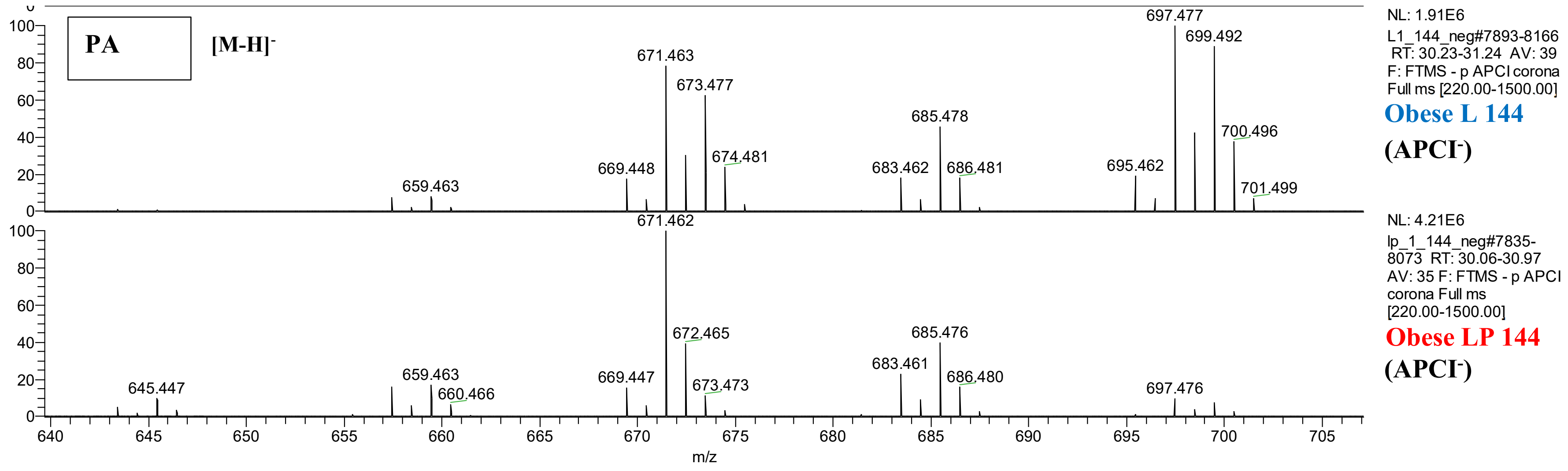

Figure S7. PA mass spectra in obese L and obese LP at 144 hours.

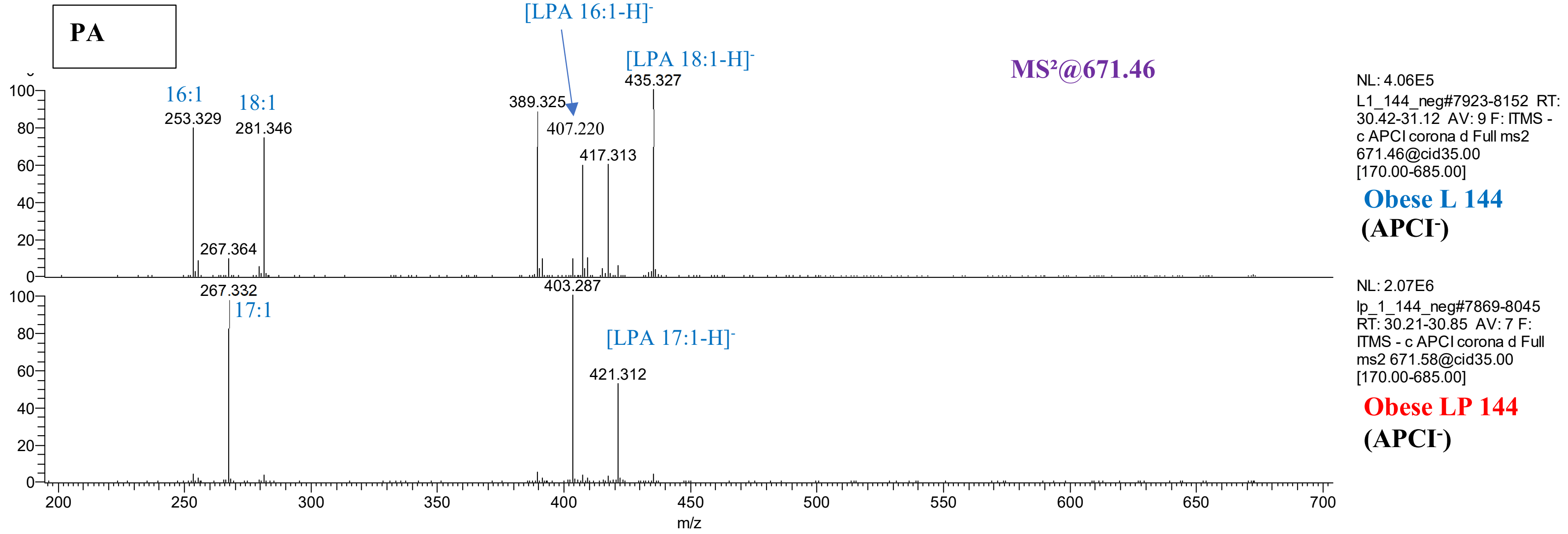

Figure S8. MS² spectra of ions @671.46 of PA in obese L and obese LP at 144 hours.

Table S3. Identification of molecular species present in PA. The m/z observed were imput in the Lipid Maps database (column 1). The corresponding matched mass are indicated in column 2. The delta mass, the name, the formula and the corresponding Ion are indicated in column 3 to 6, respectively. In column 7 and 8, the main molecular species identified according to their name and MS^2 are indicated for obese L and obese LP, respectively.

Input Mass	Matched Mass	Delta	Name	Formula	Ion	L	LP
645.4470	645.4501	0.0031	PA 32:1	C35H67O8P	$[M-H]^-$		15:0/17:1
657.4481	657.4501	0.002	PA 33:2	C36H67O8P	$[M-H]^-$	17:1/16:1	17:1/16:1
659.4634	659.4657	0.0023	PA 33:1	C36H69O8P	$[M-H]^-$	17:1/16:0 & 18:1/15:0	16:0/17:1
669.4482	669.4501	0.0019	PA 34:3	C37H67O8P	$[M-H]^-$	18:2/16:1	17:1/17:2
671.4634	671.4657	0.0023	PA 34:2	C37H69O8P	$[M-H]^-$	18:1/16:1	17:1/17:1
673.4781	673.4814	0.0033	PA 34:1	C37H71O8P	$[M-H]^-$	18:1/16:0	17:1/17:0 & 16:0/18:1
683.463	683.4657	0.0027	PA 35:3	C38H69O8P	$[M-H]^-$	18:2/17:1	17:1/18:2
685.4783	685.4814	0.0031	PA 35:2	C38H71O8P	$[M-H]^-$	18:1/17:1	17:1/18:1
695.4627	695.4657	0.003	PA 36:4	C39H69O8P	$[M-H]^-$	18:2/18:2	18:2/18:2 & 17:2/19:2
697.4778	697.4814	0.0036	PA 36:3	C39H71O8P	$[M-H]^-$	18:1/18:2	18:2/18:1
699.4924	699.497	0.0046	PA 36:2	C39H73O8P	$[M-H]^-$	18:1/18:1	18:1/18:1

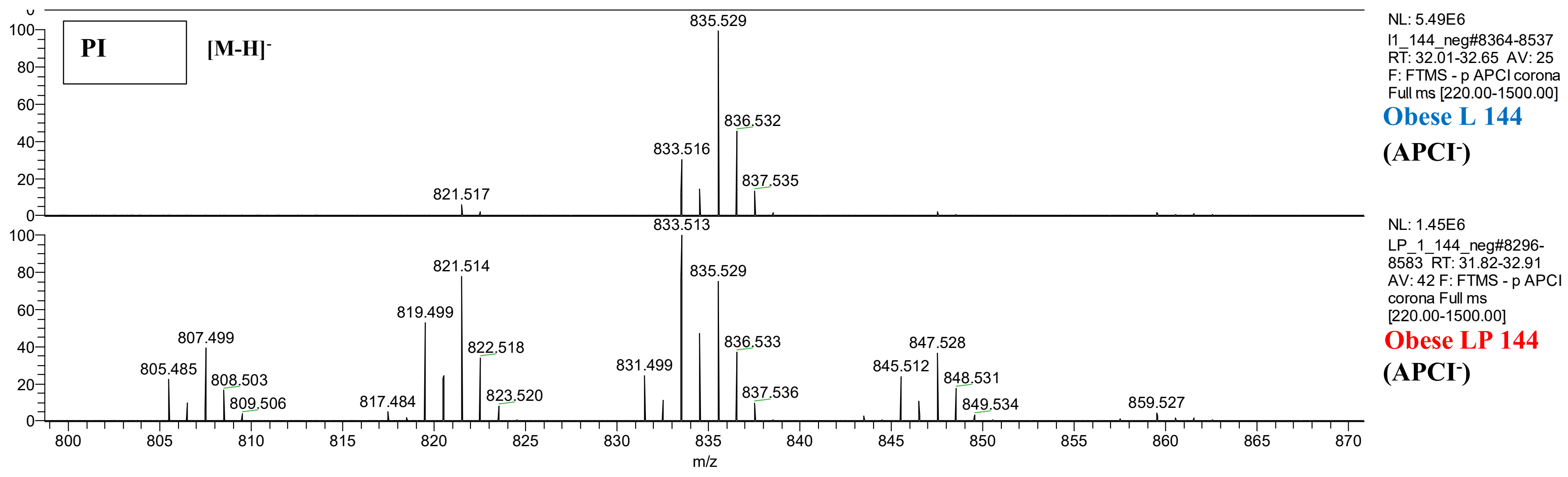

Figure S9.: PI mass spectra in obese L and obese LP at 144 hours.

109

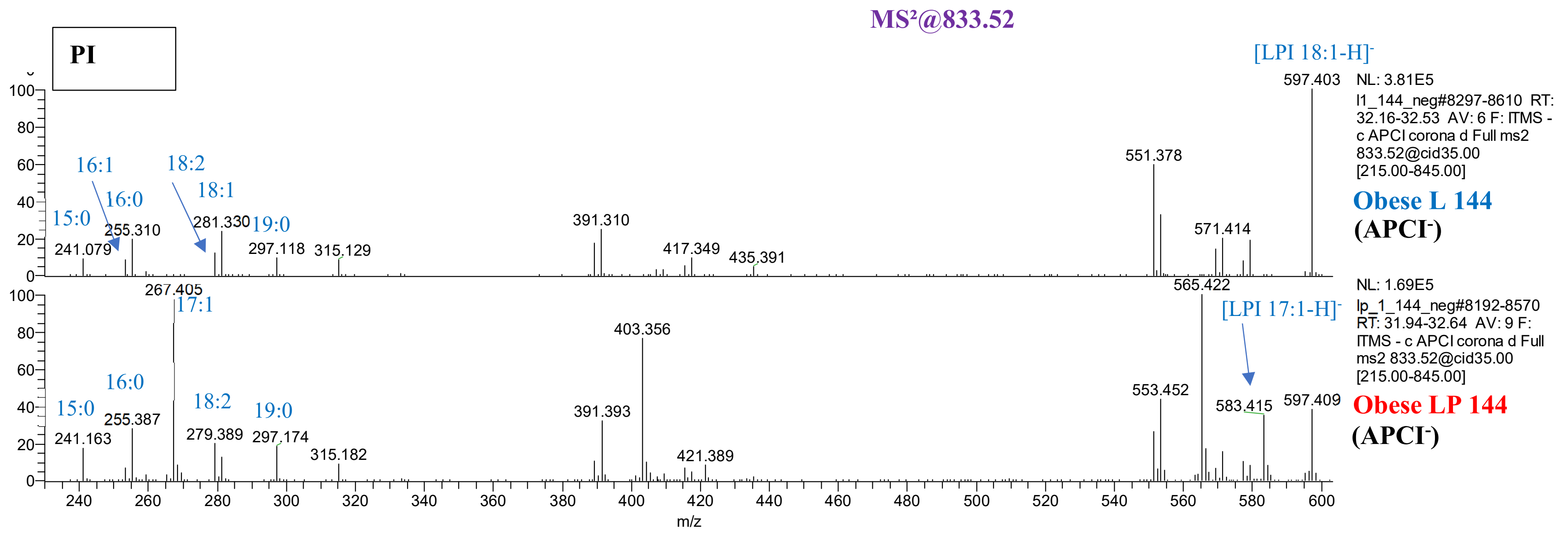

110

111

Figure S10. MS² spectra of ions @833.52 of PI in obese L and obese LP at 144 hours. 112

113

114

Table S4. Identification of molecular species present in PI. The m/z observed were imput in the Lipid Maps database (column 1). The corresponding matched mass are indicated in column 2. The delta mass, the name, the formula and the corresponding Ion are indicated in column 3 to 6, respectively. In column 7 and 8, the main molecular species identified according to their name and MS^2 are indicated for obese L and obese LP, respectively.

Input Mass	Matched Mass	Delta	Name	Formula	Ion	L	LP
805.4846	805.4873	0.0027	PI 32:2	C41H75O13P	$[M-H]^-$		17:1/15:1
807.4993	807.5029	0.0036	PI 32:1	C41H77O13P	$[M-H]^-$		17:1/15:0
817.4839	817.4873	0.0034	PI 33:3	C42H75O13P	$[M-H]^-$		18:2/15:1
819.4992	819.5029	0.0037	PI 33:2	C42H77O13P	$[M-H]^-$		17:1/16:1 & 15:0/18:2
821.5141	821.5186	0.0045	PI 33:1	C42H79O13P	$[M-H]^-$	17:1/16:0 & 18:1/15:0	16:0/17:1 & 18:1/15:0
831.4985	831.5029	0.0044	PI 34:3	C43H77O13P	$[M-H]^-$		17:1/17:2 & 18:2/16:1
833.5133	833.5186	0.0053	PI 34:2	C43H79O13P	$[M-H]^-$	16:0/18:2 & 16:1/18:1	17:1/17:1
835.5293	835.5342	0.0049	PI 34:1	C43H81O13P	$[M-H]^-$	18:1/16:0	17:1/17:0 & 16:0/18:1
843.4977	843.5029	0.0052	PI 35:4	C44H77O13P	$[M-H]^-$		17:2/18:2
845.5124	845.5186	0.0062	PI 35:3	C44H79O13P	$[M-H]^-$	18:2/17:1	17:1/18:2
847.5279	847.5342	0.0063	PI 35:2	C44H81O13P	$[M-H]^-$	18:1/17:1	17:1/18:1
849.5435	849.5499	0.0064	PI 35:1	C44H83O13P	$[M-H]^-$	18:2-18:2	17:1/18:0 & 18:1/17:0
857.5125	857.5186	0.0061	PI 36:4	C45H79O13P	$[M-H]^-$		18:2/18:2

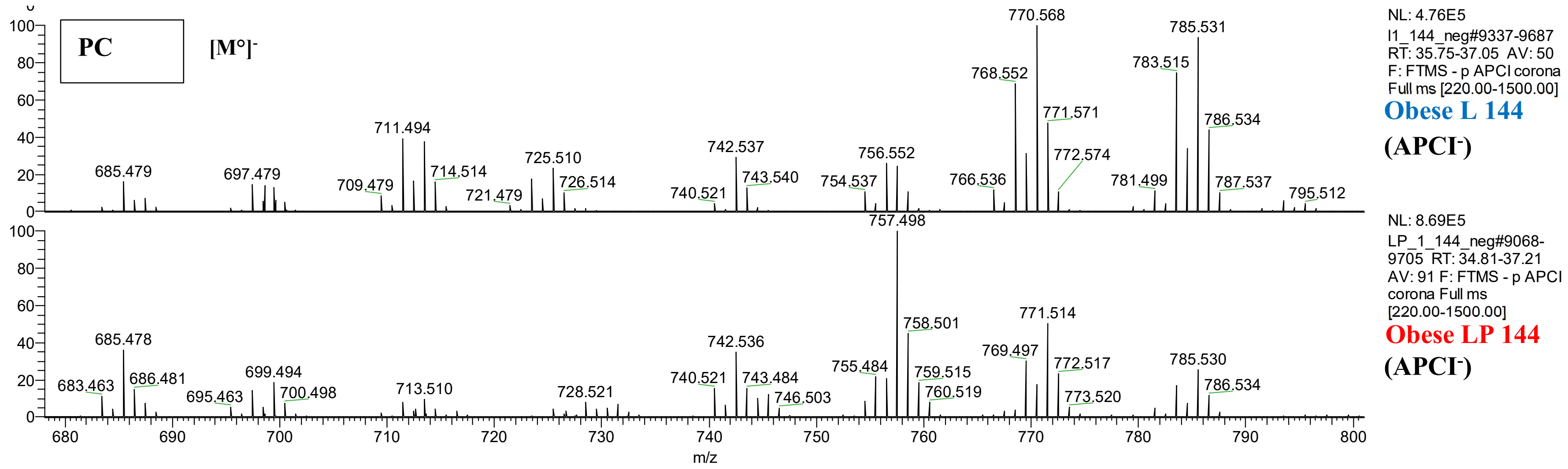

122

Figure S11. PC mass spectra in obese L and obese LP at 144 hours. 123

124

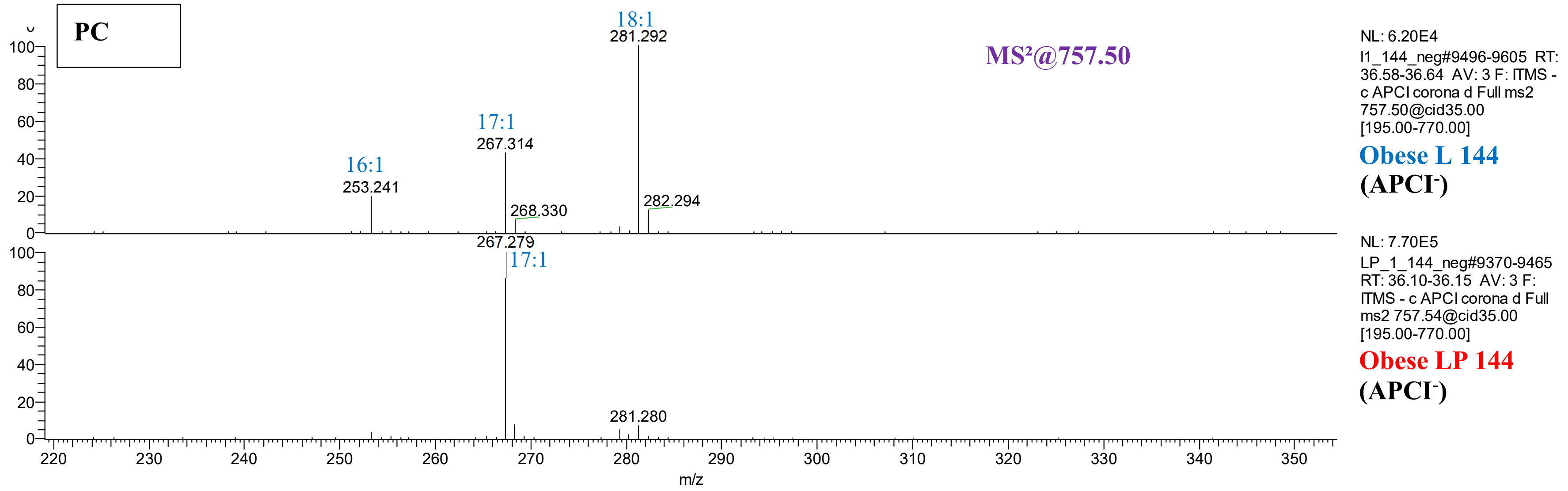

Figure S12. MS² spectra of ions @757.50 of PC in obese L and obese LP at 144 hours.

Table S5. Identification of molecular species present in PC. Manual identification of the observed m/z (column 1). The corresponding PC species and the main molecular species identified according to their MS^2 are indicated for obese L and obese LP in column 4 and 5, respectively.

LIPID MAPS®, does not allow a PC search via ions $[M°]^-$.

Mass	Name	Ion	L	LP
729.4678	PC(32:2)	$[M°]^-$		
731.4818	PC(32:1)	$[M°]^-$		15:0/17:1
741.4678	PC(33:3)	$[M°]^-$		
743.4828	PC(33:2)	$[M°]^-$	17:1/16:1	17:1/16:1
745.4974	PC(33:1)	$[M°]^-$	17:1/16:0 & 18:1/15:0	16:0/17:1
755.4829	PC(34:3)	$[M°]^-$	18:2/16:1	17:1/17:2
757.4978	PC(34:2)	$[M°]^-$	18:1/16:1	17:1/17:1
759.5132	PC(34:1)	$[M°]^-$	18:1/16:0	17:1/17:0 & 16:0/18:1
767.4825	PC(35:4)	$[M°]^-$		
769.4972	PC(35:3)	$[M°]^-$	18:2/17:1	17:1/18:2
771.5134	PC(35:2)	$[M°]^-$	18:1/17:1	17:1/18:1
773.5298	PC(35:1)	$[M°]^-$		
781.4962	PC(36:4)	$[M°]^-$	18:2/18:2	18:2/18:2 & 17:2/19:2
783.5121	PC(36:3)	$[M°]^-$	18:1/18:2	18:2/18:1
785.5296	PC(36:2)	$[M°]^-$	18:1/18:1	18:1/18:1

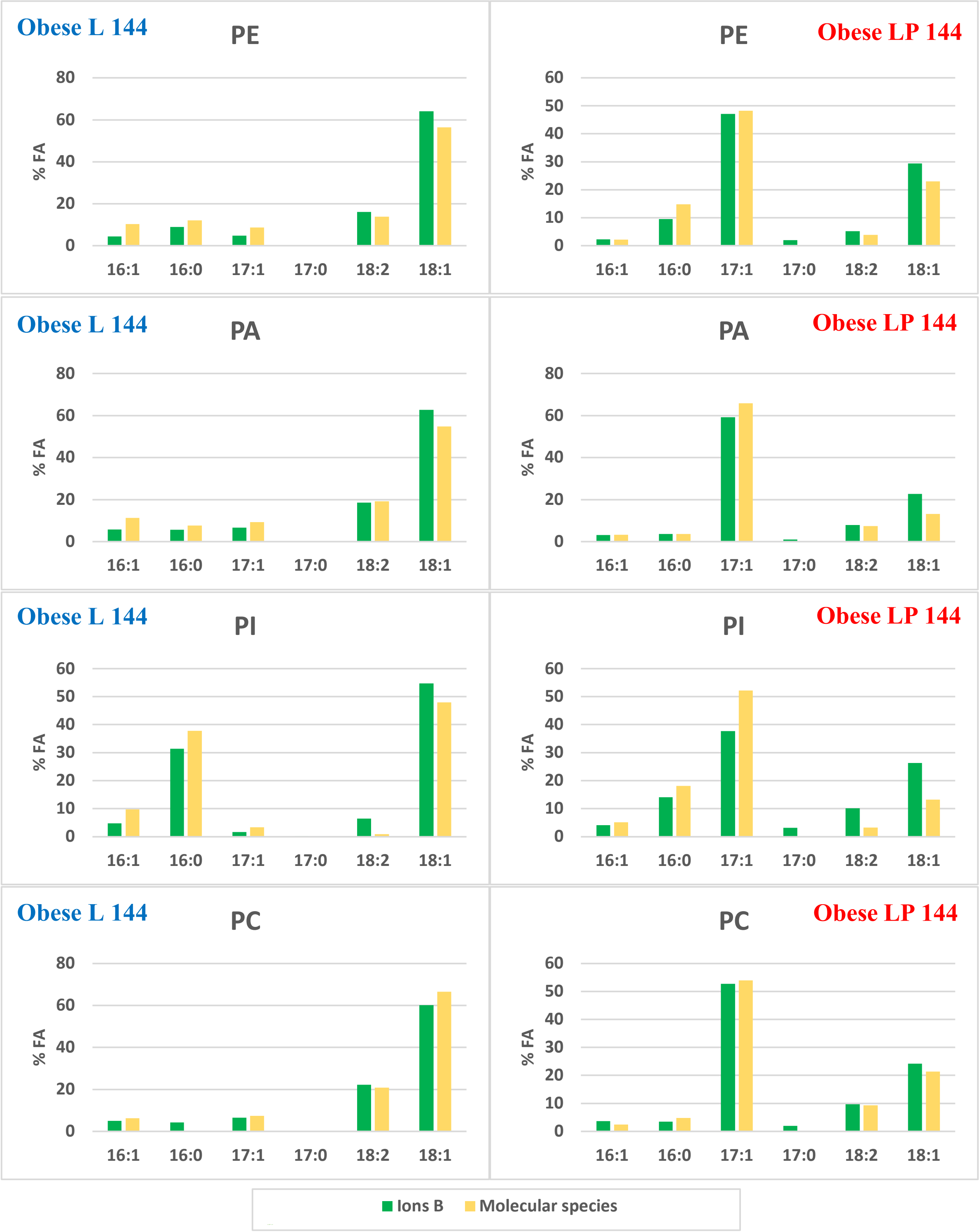

Figure S13. Obese-L and obese-LP (at 144 hours) FA distribution of PLs classes calculated from Ion B intensities (green bars) or from molecular species intensities (yellow bars).

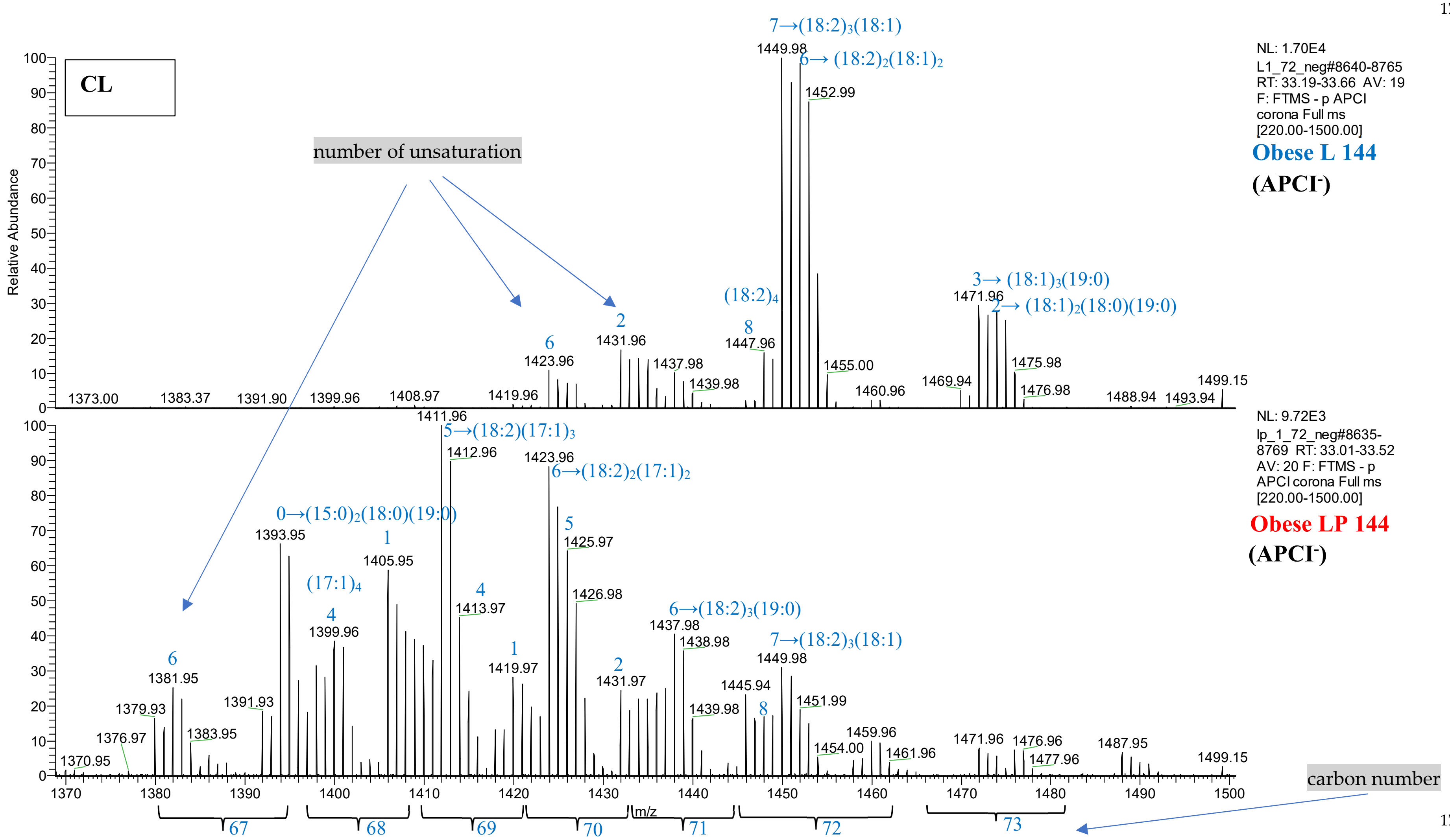

Figure S14. CL mass spectra in obese L and obese LP at 144 hours.

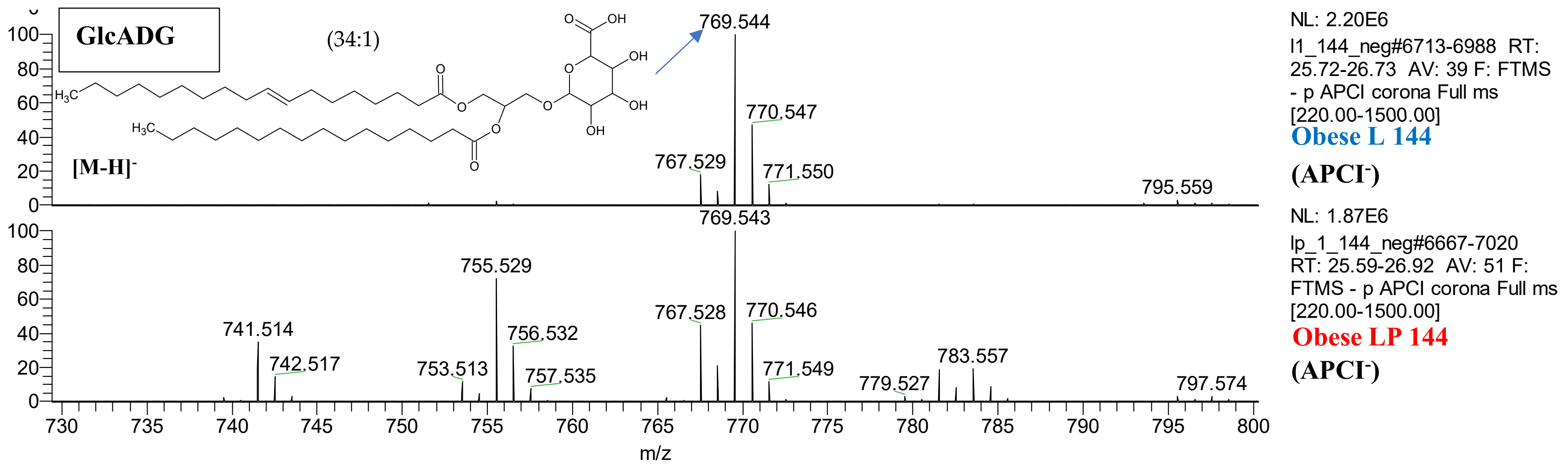

Figure S15. GlcADG mass spectra in obese L and obese LP at 144 hours.

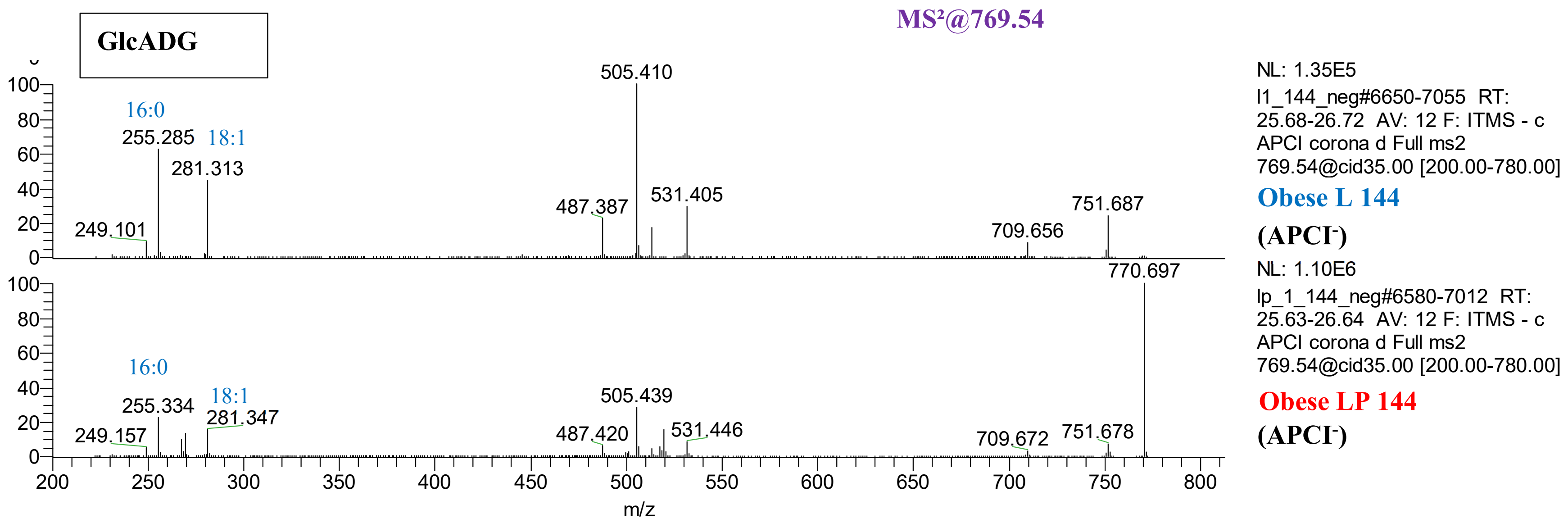

Figure S16. MS² spectra of ions @769.54 of GlcADG in obese L and obese LP at 144 hours.

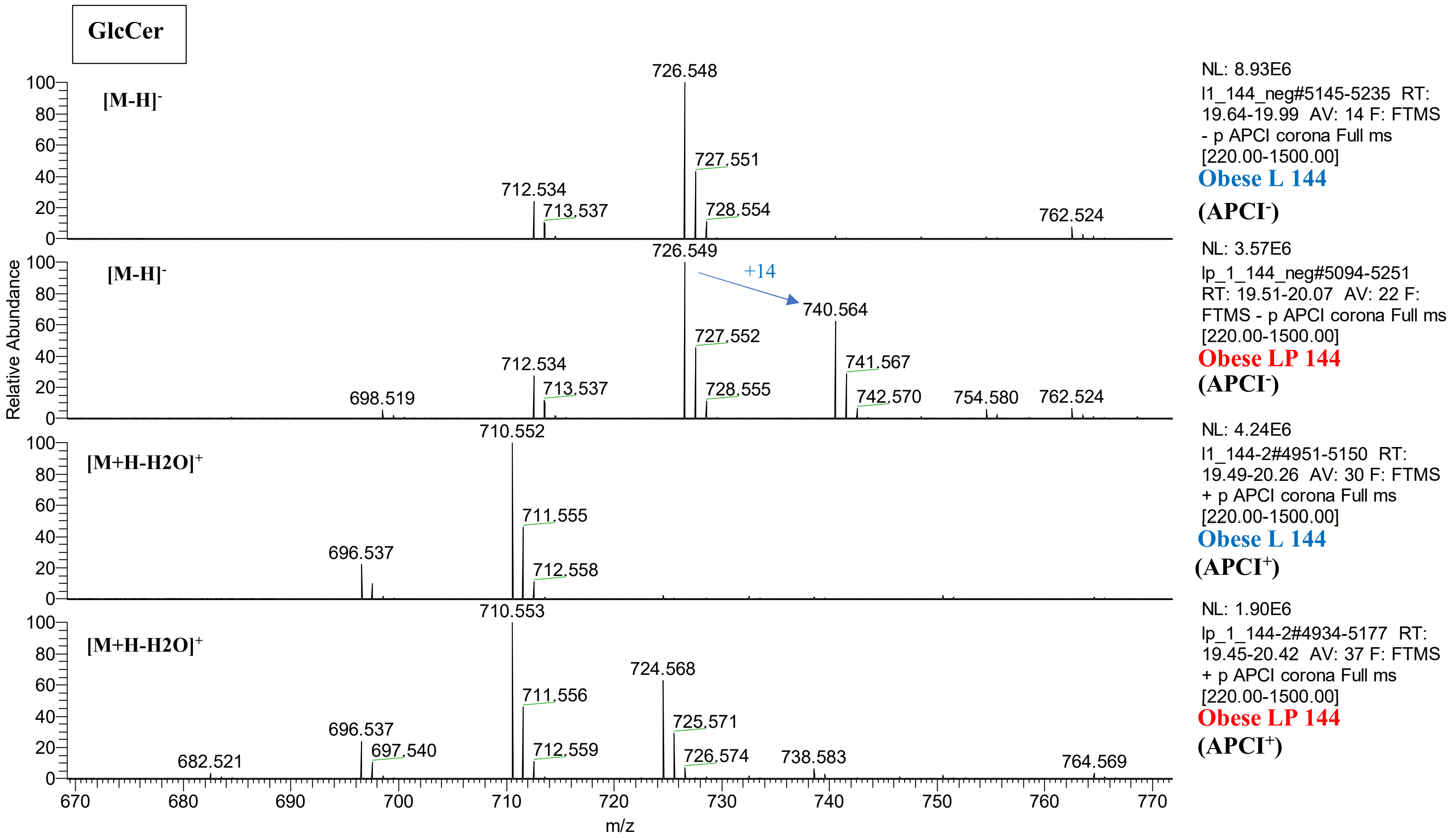

187

Figure S17: GlcCER mass spectra in obese L and obese LP at 144 hours. 188

189

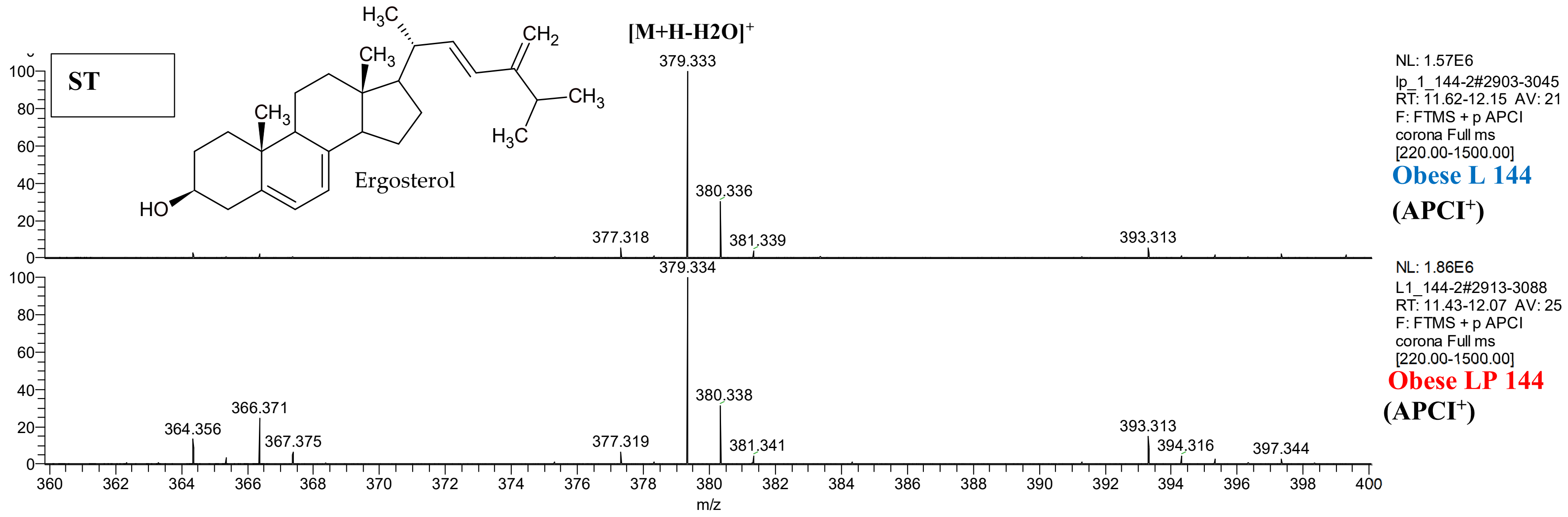

190

Figure S18. ST Mass spectra of Ergosterol in obese L and obese LP at 144 hours. 191
